# Single cell profiling of female breast fibroadenoma reveals distinct epithelial cell compositions and therapeutic targets

**DOI:** 10.1038/s41467-023-39059-3

**Published:** 2023-06-16

**Authors:** Zhigang Chen, Yi Zhang, Wenlu Li, Chenyi Gao, Fengbo Huang, Lu Cheng, Menglei Jin, Xiaoming Xu, Jian Huang

**Affiliations:** 1 Department of Breast Surgery (Surgical Oncology), Second Affiliated Hospital, Zhejiang University School of Medicine, 88 Jiefang Road, Hangzhou, Zhejiang China; 2 Key Laboratory of Tumor Microenvironment and Immune Therapy of Zhejiang Province, Hangzhou, China; 3 Cancer Centre, Zhejiang University, Hangzhou, Zhejiang China; 4 Cancer Institute, Key Laboratory of Cancer Prevention and Intervention, Ministry of Education, The Second Affiliated Hospital, Zhejiang University School of Medicine, Hangzhou, Zhejiang China; 5 Departments of Radiology, Massachusetts General Hospital, Harvard Medical School, Boston, MA 02129 USA; 6 Department of Pathology, The Second Affiliated Hospital, Zhejiang University School of Medicine, Hangzhou, Zhejiang China

**Keywords:** Translational research, Cancer therapy, Cancer

## Abstract

Fibroadenomas (FAs) are the most common breast tumors in women. No pharmacological agents are currently approved for FA intervention owing to its unclear mechanisms and a shortage of reproducible human models. Here, using single-cell RNA sequencing of human FAs and normal breast tissues, we observe distinct cellular composition and epithelial structural changes in FAs. Interestingly, epithelial cells exhibit hormone-responsive functional signatures and synchronous activation of estrogen-sensitive and hormone-resistant mechanisms (*ERBB2*, *BCL2* and *CCND1* pathways). We develop a human expandable FA organoid system and observe that most organoids seem to be resistant to tamoxifen. Individualized combinations of tamoxifen with ERBB2, BCL2 or CCND1 inhibitors could significantly suppress the viability of tamoxifen-resistant organoids. Thus, our study presents an overview of human FA at single-cell resolution that outlines the structural and functional differences between FA and normal breast epithelium and, in particular, provides a potential therapeutic strategy for breast FAs.

## Abstract (web summary)

Fibroadenoma occurrence is linked to increased long-term risk of breast carcinoma, however, the lack of models has made understanding this mechanism unclear. Here, the authors use single cell RNA-seq to identify dysregulation of epithelial cells and develop organoid models to study treatment resistance.

## Introduction

Fibroadenomas (FAs) of the breast are fibroepithelial tumors that are most common in adolescent women, although they may be diagnosed at any age^1^. Approximately 10% of the female population suffers from breast fibroadenoma in their lifetime. Apart from psychological distress and reduced quality of life among those diagnosed, FAs are associated with an increased long-term risk of developing breast cancer^2^. Surgical excision is the most effective intervention for FAs, but undesirable scarring or extensive ductal damage can occur^1^. Many patients have multiple lesions, making it difficult to eradicate all lesions. Nonsurgical intervention options for these patients are urgently needed.

FAs are clinically recognized to be hormone dependent^3^; however, the therapeutic efficacy of the estrogen receptor modulator tamoxifen in previous clinical trials has not been ideal^4,5^. Thus, dissecting the pathological mechanism underlying human breast FAs is crucial for developing more effective therapies. Although intensive sequencing studies have revealed the presence of highly recurrent mutations and the genomic landscapes of FAs^6–9^, the mechanisms underlying the transcriptional differences in epithelial and stromal cells between FAs and normal breast tissues remain largely unknown, at least in part owing to the complexity of the disease and shortage of reproducible human models.

Here, we leveraged high-throughput single-cell RNA-seq analysis to generate a detailed transcriptomic atlas of human breast FAs and normal tissues and to explore the functional differences and pathway alterations in breast FAs. Moreover, we established an expandable patient-derived FA organoid platform and conducted a systematic survey of pathway inhibitors to test the therapeutic response and suggest individualized treatments for FAs.

## Results

### Single-cell transcriptome profiling identified cell composition and epithelial structural features in FAs

**Fig. 1:**
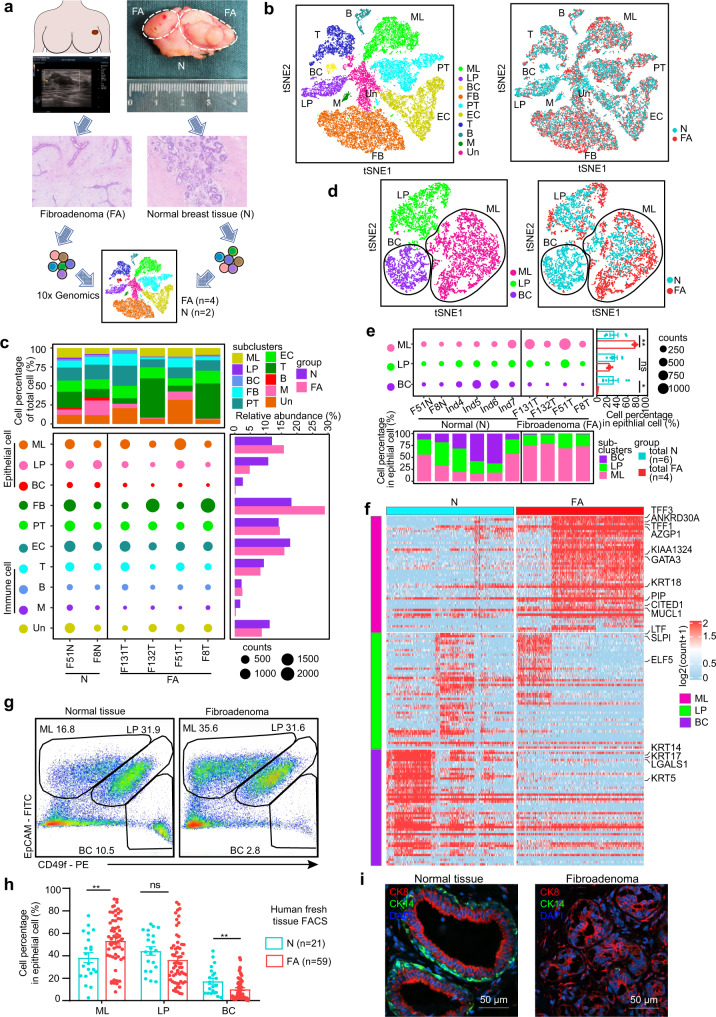
Single-cell transcriptome atlas of human breast fibroadenomas and adjacent normal tissues. **a** Study overview. Human breast fibroadenoma tissues (FA) and adjacent normal tissues (N) were collected from patients diagnosed with FA via ultrasound after the operation, verified by a pathologist, and subjected to the single-cell assays shown. **b** t-SNE analysis of 28,101 single-cell transcriptomes (17,776 from fibroadenoma and 10,325 from adjacent normal tissues). 10 cell clusters are shown: mature luminal cells (ML), luminal progenitor cells (LP), basal cells (BC), fibroblasts (FB), endothelial cells (EC), pericytes (PT), T cells (T), B cells (B), myeloid cells (M) and unknown cells (Un). **c** Cell composition in each sample (top); cell counts of different cell subsets in each sample (middle); relative abundance of different cell subsets (right). **d** t-SNE map of 6346 single-cell transcriptomes of mature luminal cells (ML), luminal progenitor cells (LP), and basal cells (BC) (3173 from fibroadenoma and 3173 from normal tissues). **e** Cell counts of different cell subsets in each sample (top); cell percentage in total cells of the fibroadenomas (*n* = 4) and normal tissues (*n* = 6) (right); Each dot represents one patient. Data are shown as mean +/− s.e.m. **p = 0.0033 (ML), ns *p* = 0.1636 (LP), *p = 0.0286 (BC), unpaired two-tailed Student’s t test; cell percentage in total cells of each sample (bottom). **f** Heatmap of log-normalized gene expression (log2(count + 1)) of the epithelium subset genes from fibroadenoma (FA) and normal tissues (N). **g** FACS analysis of epithelial cell subtypes in fibroadenoma and normal tissues gated at CD45-CD31- cells. ML (mature luminal cells, EpCAM^+^CD49f^lo^), LP (luminal progenitor cells, EpCAM^+^CD49f^hi^), and BCs (basal cells, EpCAM^-^CD49f^hi^). **h** Quantification of epithelial cell subtypes in human fibroadenoma fresh tissue (*n* = 59) and normal tissues (*n* = 21). Data are expressed as the mean +/− s.e.m. Each dot represents one patient. **p = 0.0083 (ML), ns *p* = 0.1461 (LP), **p = 0.0083 (BC), unpaired two-tailed Student’s t test. **i** Immunofluorescence staining of paraffin sections from fibroadenomas and normal tissues. Red: CK8 (luminal cells); Green: CK14 (basal cells); Blue: DAPI (nuclei). Scale bar: 50 μm. Experiment was performed with three independent replicates. Source data are provided as a Source data file.

To obtain an unbiased and comprehensive comparison of the gene expression of cell types in FAs and normal breast tissue, single-cell RNA sequencing was performed with 4 fresh breast FAs and 2 paired adjacent normal tissues from human female individuals who were diagnosed with typical FAs (Fig. 1a, Supplementary Fig. 1, Supplementary Data 1). A total of 28,101 single-cell transcriptomes (17,776 from fibroadenoma and 10,325 from normal) with 23,437 genes were detected (Supplementary Data 2). The cells were assigned to 10 cell clusters by their distinct gene expression signatures: mature luminal cells (ML), luminal progenitor cells (LP) and basal cells (BC), fibroblasts (FB), endothelial cells (EC), pericytes (PT), T cells (T), B cells (B), and myeloid cells (M) as well as unknown cells (Un) (Fig. 1b, Supplementary Fig. 2, Supplementary Data 2).

**Fig. 2:**
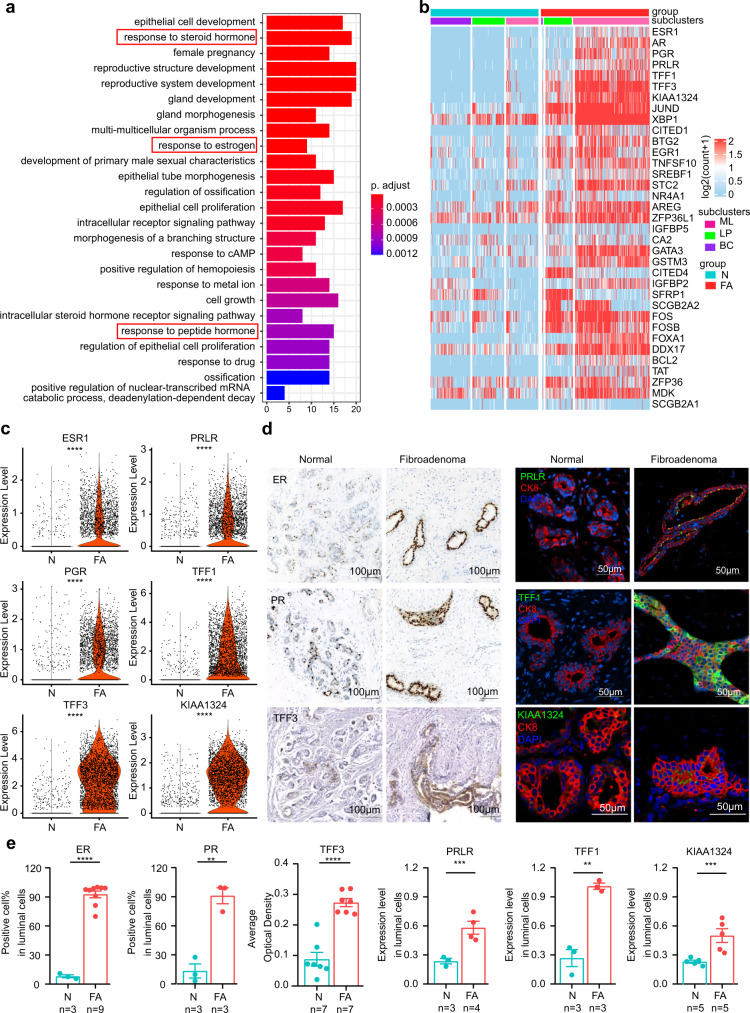
Hormone-responsive functional signatures in the epithelium of human breast fibroadenomas. **a** GO analysis of differentially expressed genes in epithelial cells between fibroadenoma and normal tissue. one-sided, hypergeometric test and adjusted by Benjamini-Hochberg correction. **b** Heatmap of hormone-responsive gene expression between fibroadenomas (FA) and normal tissue (N). **c** Violin plots showing the expression of genes from (**b**) in the epithelial cells (N: *n* = 3173; FA: *n* = 3173) of fibroadenomas and normal tissues from the single-cell analysis. ****p < 0.0001, two-sided, Wilcoxon test. **d** Immunohistochemical staining or immunofluorescence staining of genes in (**c**). Scale bar: 100 μm (IHC), 50 μm (IF). **e** Scatter diagram of immunohistochemical staining or immunofluorescence staining in (**d**) between fibroadenomas (FA) and normal tissues (N). Data are expressed as the mean +/− s.e.m. Each dot represents one patient. ****p = 0.0000001 (ER, N: *n* = 3; FA: *n* = 9), **p = 0.0021 (PR, N: *n* = 3; FA: *n* = 3), ****p = 0.000012 (TFF3, N: *n* = 7; FA: *n* = 7), ***p = 0.0008 (PRLR, N: *n* = 3; FA: *n* = 4), **p = 0.0014 (TFF1, N: *n* = 3; FA: *n* = 3), ***p = 0.0005 (KIAA1324, N: *n* = 5; FA: *n* = 5), unpaired two-tailed Student’s t test. Source data are provided as a Source data file.

The cell type composition in FAs was different from that in paired adjacent normal breast tissues (Fig. 1c). Among stromal cells, fibroblasts increased significantly in FAs (Fig. 1c, Supplementary Fig. 3a), while endothelial cells, pericytes and immune cells were only slightly altered (Supplementary Data 3, Supplementary Figs. 14, 15, Supplementary Data 12). Differentially expressed gene (DEG) analysis identified 104 upregulated DEGs and 159 downregulated DEGs in fibroblasts of FAs (Supplementary Fig. 14a, Supplementary Data 3). Gene Ontology (GO) enrichment analysis revealed that upregulated genes were mainly associated with the regulation of actin filament-based processes and actin cytoskeleton organization (Supplementary Fig. 15a). Analysis of the proliferating genes (*MKI67, PCNA*) and immunohistochemical staining of KI67 in fibroblasts revealed that the increase in fibroblasts may not be caused by higher proliferation (Supplementary Fig. 3b, c). Fibroadenoma has been reported to be hormone sensitive. Therefore, we aimed to determine whether the increase in fibroblasts was hormone induced. We further investigated the expression of hormone receptors (*ER, PR, PRLR*) in stromal cells, including pericytes, fibroblasts, endothelial cells, and immune cells. Surprisingly, we observed that stromal cells in fibroadenoma barely expressed hormone receptors (*ER, PR, PRLR*), while the epithelium expressed high levels of hormone receptors (Supplementary Fig. 3d). Immunohistochemical staining showed that hormone receptors (ER, PR, PRLR) were mainly expressed in the epithelium rather than stromal cells (Fig. 2d, Supplementary Fig. 3e).

We determined whether increased fibroblasts were recruited by epithelial cells in fibroadenomas. We isolated primary epithelial and fibroblast cells from patient fibroadenoma samples and paired normal tissues and set up an epithelial and fibroblast coculture system (Supplementary Fig. 3f, g). The epithelial and fibroblast coculture system demonstrated that fibroblasts in normal breast tissue could be recruited by the FA epithelium (Supplementary Fig. 3f, g). This evidence indicated the important role of the epithelium in the formation of FAs.

Our scRNA data showed that the epithelial cell composition in FAs changed remarkably and was distinguished from normal breast epithelial cells with expanded mature luminal cells (1.3-fold) and reduced basal cells (9-fold) (Fig. 1c, Supplementary Data 2). To further characterize the cell composition of the epithelium in FAs, we clustered the epithelial cell compartments from our 4 human FAs with 2 paired adjacent normal tissues plus 4 previously reported primary human breast epithelial cells isolated from reduction mammoplasties from the GEO database (GSE113197)^10^. We found that the basal cell population was almost completely diminished in FAs, whereas mature luminal cells were markedly enriched (Fig. 1d, e, Supplementary Data 4, Supplementary Fig. 4). In addition, the gene expression profile suggested that luminal cell marker genes, including *GATA3* and *KRT18*, were upregulated, while basal cell marker genes (*KRT14, KRT17, LGALS1, KRT5*) were downregulated in FAs (Fig. 1f, Supplementary Data 5). To confirm this, we performed FACS separation based upon combinations of EpCAM and CD49f expression to characterize epithelial subsets from 59 human breast FA fresh tissues and 21 normal breast tissues^11^ (Fig. 1g, h, Supplementary Fig. 5, Supplementary Data 6). Indeed, the ratio of basal cells (CD49f^hi^EpCAM^-^) in FAs was significantly decreased, and mature luminal cells (CD49f^lo^EpCAM^+^) were increased compared to normal tissues (Fig. 1h, Supplementary Fig. 5). A normal mammary gland consists of an outer layer of basal cells and inner luminal cells^12^, and the loss of basal cells in FAs indicates a change in the bilayer structure. Immunofluorescence staining further revealed a loss of basal cells (CK14) and a decrease in the double lumen structure in FAs (Fig. 1i). Thus, these results indicated that the cell composition and structural characteristics of FAs were distinct from those of the normal breast epithelium.

### Epithelium in FAs displaying hormone-responsive signatures

We next asked whether functional differences existed in the epithelial cell population in FAs. We compared the DEGs (|avg log2FC| > 0.5 and *p* value adjusted <0.05) of epithelial cells. We identified 381 upregulated DEGs and 485 downregulated DEGs in epithelial cells (Supplementary Data 7). Moreover, GO analysis of enriched genes (more than 1.5-fold; adjusted *p* < 0.01) showed that epithelial cells in FAs were overrepresented in categories related to the response to hormones (Fig. 2a, Supplementary Data 8). The enriched genes included hormone receptors such as *ESR1, AR, PGR,* and *PRLR* as well as hormone-responsive lineage-specific transcription factors such as *FOXA1*, *GATA3*, and *FOS* (Fig. 2b). Among these enriched genes associated with the hormone response, the fold change in *TFF1* and *TFF3*, which are regulated by estrogen and *KIAA1324* (also known as estrogen-induced gene 121, *EIG121*), was the most significant (Fig. 2c). Immunofluorescence and immunohistochemical staining further confirmed these hormone-related protein expression patterns in patients with FAs (Fig. 2d, e). We then compared the DEGs of 3 different epithelial cell types, including mature luminal cells, luminal progenitor cells, and basal cells (Supplementary Fig. 7, Supplementary Data 7). 397 upregulated DEGs and 638 downregulated DEGs were identified in mature luminal cells (Supplementary Fig. 7a, Supplementary Data 7). GO enrichment analysis revealed that upregulated genes were mainly associated with the hormone response signaling pathway (Supplementary Fig. 8a, Supplementary Data 9). Given that mature luminal cells were substantially expanded in FAs and that luminal cells are physiologically known to respond to endocrine signals or produce milk^13^, we analyzed hormone-sensing genes (*ESR1, PGR, PRLR*, and *CITED1*) and milk-synthesis genes (*CSN3, LTF, OLAH*) in FAs. The results showed that epithelial cells in FAs were mainly hormone sensitive (Supplementary Fig. 6). 232 upregulated DEGs and 546 downregulated DEGs were identified in luminal progenitor cells (Supplementary Fig. 7b, Supplementary Data 7). GO enrichment analysis revealed that upregulated genes were mainly associated with epithelial cell proliferation (Supplementary Fig. 8b, Supplementary Data 9). We also identified 230 upregulated DEGs and 305 downregulated DEGs in basal cells (Supplementary Fig. 7c, Supplementary Data 7). GO enrichment analysis revealed that upregulated genes were mainly associated with the regulation of gland development (Supplementary Fig. 8c, Supplementary Data 9). These results demonstrated that epithelial cells in FAs displayed hormone-responsive functional signatures.

### Coexistence of hormone-sensitive and hormone-resistant pathways in FAs

**Fig. 3:**
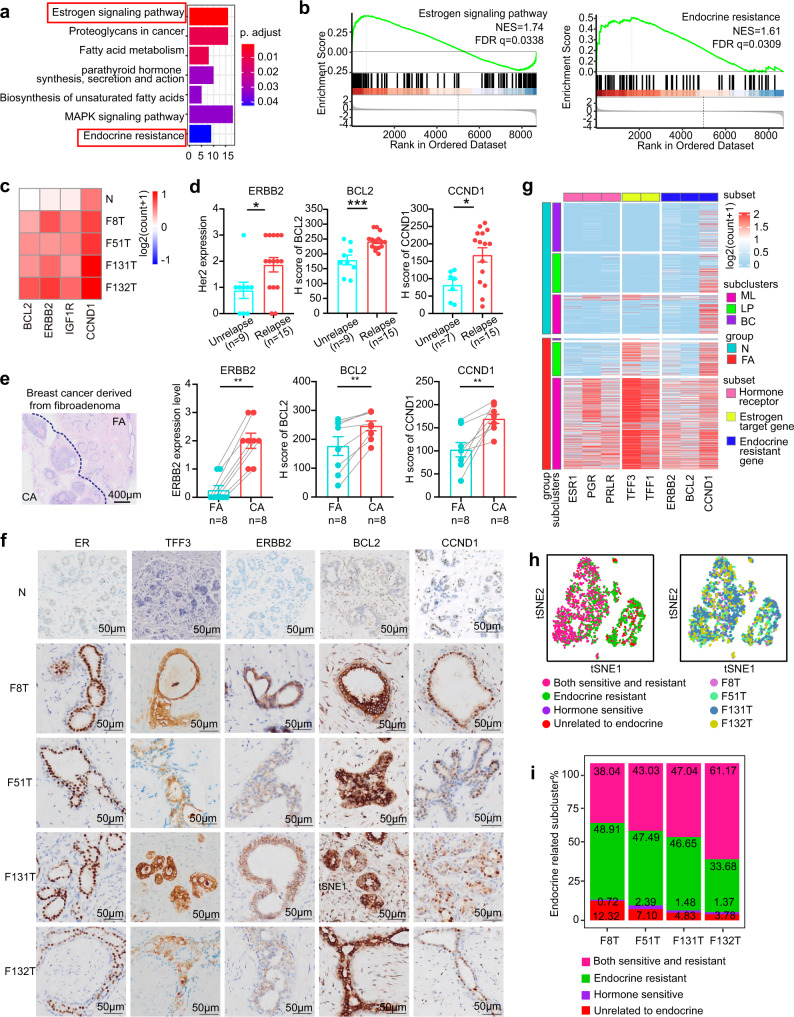
Coexistence of hormone-sensitive and -resistant pathways in the epithelium of fibroadenomas. **a** KEGG analysis of differentially expressed genes in epithelial cells between fibroadenoma and normal tissue. Pathway analysis was determined by a hypergeometric test, one-sided and adjusted *p* < 0.05 was considered statistically significant. **b** GSEA of the estrogen signaling pathway and endocrine resistance pathway in epithelial cells between fibroadenoma and normal tissue. **c** Heatmap of the average expression of endocrine resistance genes from (**b**) in epithelial cells. **d** The expression of endocrine resistance genes (ERBB2, BCL2, and CCND1) in relapsed and non-relapsed fibroadenoma patients using immunohistochemical staining. Data are expressed as the mean +/− s.e.m. Each dot represents one patient. *p = 0.01306 (ERBB2, non-relapse: *n* = 9; relapse: *n* = 15), two-sided, Fisher’s test. ***p = 0.0006 (*BCL2*, non-relapse: *n* = 9; relapse: *n* = 15), *p = 0.0142 (CCND1, non-relapse: *n* = 7; relapse: *n* = 15), unpaired two-tailed Student’s t test. **e** The expression of endocrine resistance genes (ERBB2, BCL2, and CCND1) in breast cancer patients who had been pathologically proven to be derived from fibroadenoma by immunohistochemical staining. Data are expressed as the mean +/− s.e.m. Each dot represents one patient. **p = 0.00699 (ERBB2, breast cancer: *n* = 8; fibroadenoma: *n* = 8), two-sided Fisher’s test. **p = 0.0054 (*BCL2*, breast cancer: *n* = 8; fibroadenoma: *n* = 8), **p = 0.0027 (CCND1, breast cancer: *n* = 8; fibroadenoma: *n* = 8), paired two-tailed t test. **f** Immunohistochemical staining of hormone-sensitive and hormone-resistant markers in different patients. Scale bar: 50 μm. Experiment was performed with three independent replicates. **g** Heatmap of log-normalized expression (log2(count + 1)) of hormone-sensitive and resistance genes in epithelial cells between fibroadenoma and normal tissue. **h** Phenotypic distribution of epithelial cells (hormone sensitive, endocrine resistant, both sensitive and resistant, unrelated). **i** Relative proportion of endocrine-resistant epithelial cell subsets in each patient. Source data are provided as a Source data file.

How do epithelial cells regulate the hormone response in FAs? Clinical studies have reported that hormone-sensitive pathways can control FAs^3,14^. Here, we found that in addition to hormone-sensitive pathways, endocrine resistance pathways were also involved in human FAs by performing KEGG analysis (Fig. 3a, Supplementary Data 10). Gene set enrichment analysis (GSEA) provided similar results (Fig. 3b, Supplementary Data 13). Genes involved in endocrine resistance pathways primarily fell into 3 functional categories: *ERBB2/IGF1R, CCND1*, and *BCL2* (Fig. 3c, Supplementary Data 10 and 13), which have been reported to be associated with endocrine resistance^15–17^ and an increased risk of cancer recurrence in breast cancer^18,19^. We then asked whether these 3 endocrine resistance markers correlated with the recurrence of FAs and increased risk of breast cancer. We retrospectively collected data from 15 patients with FAs who experienced relapse in the same breast location within 3 years after surgery and 9 patients without relapse. As shown in Fig. 3d, the expression levels of ERBB2, CCND1, and BCL2 were significantly higher in the recurrent FA group than in the non-relapsed group. In addition, we found that the expression levels of ERBB2, CCND1 and BCL2 were positively correlated with FA recurrence (ERBB2, *P* = 0.013; CCND1, *P* = 0.004; BCL2, *P* = 0.001) (Supplementary Fig. 9a, Table 1). Furthermore, 8 patients with breast cancer that was pathologically proven to be derived from FAs were analyzed. The expression levels of ERBB2, CCND1, and BCL2 were significantly higher in the breast cancer component than in the FAs (Fig. 3e, Supplementary Fig. 9b).

**Table 1:** Protein expression of ERBB2, BCL2, and CCND1 in human breast fibroadenoma

Characteristics protein	Relapse		Tumor size		Mutiple		Bilateral	
	Yes	No	>=2 cm	<2 cm	Yes	No	Yes	No
**ERBB2 expression**								
High (2 + ) ~ (3+)	10	1	9	2	10	1	8	3
Low (−)~(+)	5	8	9	4	9	4	8	5
*p* value	0.013		0.649		0.327		0.679	
**BCL2 expression**								
High (H score > =200)	15	3	13	5	17	1	14	4
Low (H score <200)	0	6	5	1	2	4	2	4
*p* value	0.001		1		0.001		0.129	
**CCND1 expression**								
High (H score >= 150)	11	0	10	1	11	0	10	1
Low (H score <150)	4	7	8	3	6	5	6	5
*p* value	0.004		0.587		0.035		0.149	

*p* values were calculated by two-sided Fisher’s exact test.

To assess whether these endocrine resistance pathway markers coexist in the epithelial cells of each patient, we investigated the heterogeneity of human FA epithelial cells. Indeed, tSNE analysis revealed that *ERBB2*+ cells, *BCL2*+ cells, and *CCND1*+ cells could be detected from epithelial cells in each patient (Supplementary Fig. 10a). Next, we performed immunostaining in 4 human FA patients used for single-cell RNA sequencing to further validate these findings. Both hormone-sensitive markers (ER, TFF1, TFF3) and endocrine resistance markers (ERBB2, BCL2, CCND1) were detected in human patients with FAs (Fig. 3f, Supplementary Fig. 10b). In contrast, the expression levels of these proteins were lower in normal human tissue (Fig. 3f, Supplementary Fig. 10b). Unexpectedly, we found that the expression of hormone-sensitive markers (ER, TFF1, TFF3) and endocrine resistance markers (ERBB2, BCL2, CCND1) had significant heterogeneity among samples, suggesting that each subject has a distinct sensitivity to endocrine therapy.

To define the mechanisms underlying heterogeneity among samples, we first analyzed whether the coexpression of genes in these pathways existed in the same epithelial cell. The results showed that each epithelial cell had distinct expression patterns; some expressed both pathway genes, while other cells only expressed resistance markers (Fig. 3g), suggesting the presence of heterogeneous epithelial cell populations in FAs. We then reclustered the epithelial cell compartments into endocrine-sensitive (*ESR1*+*, TFF3*+) and endocrine-resistant subgroups (*ERBB2*+*, BCL2*+*, CCND1*+). As shown in Fig. 3h, the epithelial cells in FAs included four subtypes: endocrine sensitive, resistant, sensitive, and resistant coexisted and unrelated. The percentage of the endocrine-resistant subgroup showed specific heterogeneity among patient samples (Fig. 3i, Supplementary Fig. 10c, d).

### Individualized combinations of tamoxifen with CCND1, BCL2, or ERBB2 inhibitors significantly suppressed the viability of tamoxifen-resistant FA organoids

**Fig. 4:**
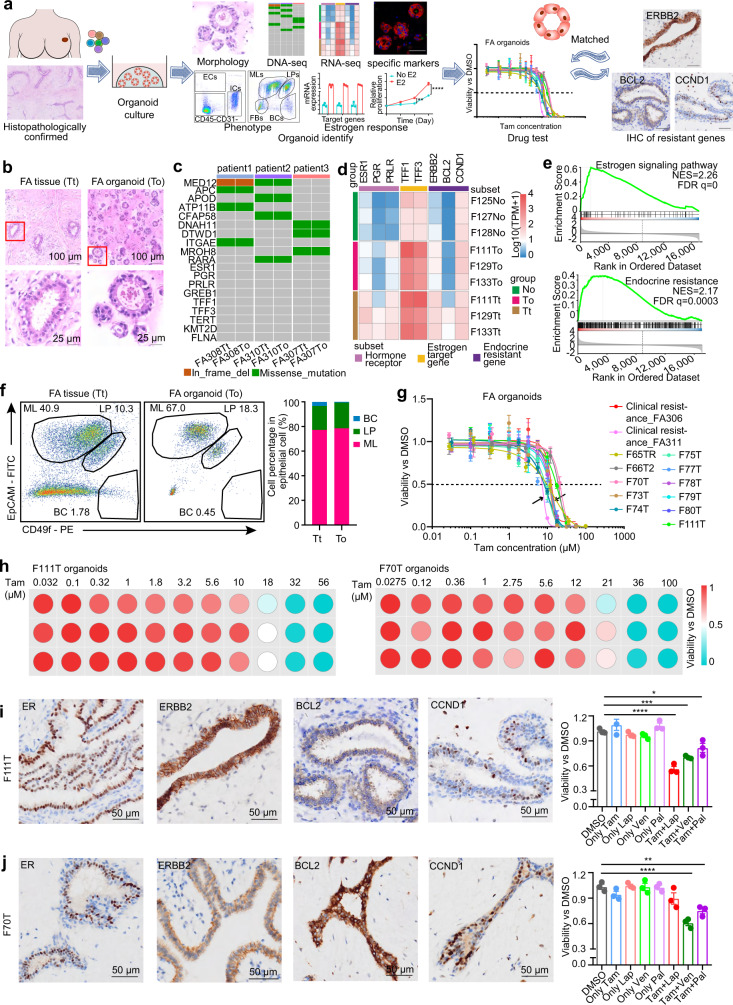
Tamoxifen combined with endocrine resistance gene inhibitors suppresses the viability of tamoxifen-resistant fibroadenoma organoids. **a** Patient-derived fibroadenoma organoid experimental setup. **b** HE staining of fibroadenoma tissue and organoids. Scale bars: 100 μm and 25 μm. Experiment was performed with three independent replicates. **c** Whole-exome sequencing of organoids and patient samples. **d** Heatmap of hormone-sensitive and hormone resistance gene signatures in fibroadenoma tissues (Tt), fibroadenoma organoids (To), and normal organoids (No) by limited RNA sequencing. **e** GSEA of the estrogen signaling pathway and endocrine resistance in fibroadenoma versus normal organoids. **f** FACS plot of epithelial cell subtypes in fibroadenoma tissue (Tt) and organoids (To) (left); quantification of epithelial cell subtypes in Tt and CD45-CD31- cells (right). **g** Drug response curves of tamoxifen in different fibroadenoma organoid lines. CellTiter-Glo® 3D Cell Viability Assay was used to measure cell viability. *n* = 3/group from three independent experiments. Data are shown as mean +/− s.e.m. **h** Heatmap of the drug sensitivity of tamoxifen in fibroadenoma organoid lines (F111T, F70T). **i** IHC of resistance pathway markers in F111T tissues (left). Scale bar: 50 μm. The drug combination test showed that F111T organoids with high ERBB2 expression were more sensitive to tamoxifen (Tam) combined with lapatinib (Lap) treatment (right). *n* = 3/group from three independent experiments. Data are expressed as the mean +/− s.e.m. ****p = 0.0000034 (DMSO vs Tam [10 μM] + Lap [10 nM]), **p = 0.00033 (DMSO vs Tam [10 μM] + Ven [5 μM]), *p = 0.022 (DMSO vs Tam [10 μM] + Pal [10 nM]), one-way ANOVA. **j** IHC of resistance pathway markers (ERBB2, BCL2, CCND1) in F70T tissues (left). Scale bar: 50 μm. The drug combination test showed that F70T organoids with high expression of CCND1 and BCL2 were more sensitive to tamoxifen (Tam) combined with palbociclib (Pal) or tamoxifen plus venetoclax (Ven) (right). *n* = 3/group from three independent experiments. Data are expressed as the mean +/− s.e.m. ***p = 0.000019 (DMSO vs Tam [10 μM] + Ven [5 μM]), **p = 0.0020 (DMSO vs Tam [10 μM] + Pal [10 nM]), one-way ANOVA. Source data are provided as a Source data file.

Because hormone-sensitive and hormone-resistant pathways were found to coexist in FAs, we explored the possibilities of developing individualized endocrine therapies for patients with FAs. To date, no FA models have been reported; thus, we developed an expandable organoid platform derived from 39 FA patients (Fig. 4a, Supplementary Data 1, Supplementary Fig. 11a). The histological features of these organoids closely resembled the original FA epithelium (Fig. 4b). To verify that organoids retain fidelity to their originating tumors, we performed whole-exome sequencing of organoids and patient tissue samples. The results showed that organoids generally retained the genomic structure of the human fibroadenoma (*MED12, APC* genomic mutations) as patient samples (Fig. 4c). RNA sequencing showed similar transcriptional changes of hormone-sensitive and hormone-resistant markers between organoids and FAs relative to normal epithelium (Fig. 4d, Supplementary Fig. 19). Hormone-sensitive markers (*ESR1, TFF1, TFF3*) and resistance pathway markers (*BCL2, CCND1, ERBB2*) were also upregulated in FA organoids compared to normal breast organoids (Fig. 4d). GSEA also revealed positive enrichment of hormone-sensitive and hormone-resistant pathways in human FA organoids (Fig. 4e, Supplementary Fig. 12a–c, Supplementary Data 14). Immunofluorescence data revealed that most of the organoids were CK8+ luminal cells that secreted KIAA1324 and AZGP1, mimicking the original FA tissues (Supplementary Fig. 11c). Moreover, FACS further demonstrated that the epithelial cells in human FA-derived organoids had almost the same cell composition as the original FAs (Fig. 4f, Supplementary Fig. 11b). To verify the hormone-responsive functional characteristics, FA-derived organoids were treated with 10 nM estradiol (E2). The mRNA expression of ER-response markers (*GREB1, TFF1, TFF3*) was significantly upregulated after E2 treatment (Supplementary Fig. 11d, e, Supplementary Data 11). Immunostaining of TFF1 showed an obvious increase in organoids treated with 10 nM E2 (Supplementary Fig. 11f). In addition, the growth of FA-derived organoids was stimulated by E2 (Supplementary Fig. 11g). These data indicated that FA-derived organoids were hormone-responsive. These findings provide evidence to support that organoids are an accurate representation of human FAs and can be used to develop individualized endocrine therapy.

Organoids derived from 13 human FA patients were treated with tamoxifen, a competitor for binding to the estrogen receptor. Heterogeneity in the response to tamoxifen was found among these organoids from different patients (Fig. 4g, Supplementary Fig. 13a). FA organoids derived from 2 clinically proven tamoxifen-resistant patients (FA306 and FA311) showed that the IC50 of tamoxifen was approximately 7–12 µM. The IC50 of most organoids was greater than that of organoids derived from clinically proven tamoxifen-resistant patients. The other two organoids from F111T and F70T patients showed more resistance to tamoxifen, as the IC50 of tamoxifen was nearly 20 µM (Fig. 4g).

To determine whether the presence of endocrine resistance markers has a causal impact on estrogen response markers, we assessed the effect of endocrine-resistant gene (ERBB2, BCL2, and CCND1) inhibitors on estrogen response markers in FA organoids. The CDK4/6 inhibitor palbociclib, the BCL2 inhibitor venetoclax, and the ERBB2 inhibitor lapatinib did not change the transcriptional expression of estrogen receptors (*ER, PR*) or estrogen response markers (*GREB1 TFF1, TFF3*) in FA organoids (Supplementary Fig. 16). These results indicated that hormone-sensitive pathways seemed to be independent of endocrine-resistant markers (ERBB2, BCL2, and CCND1) in FAs and that in targeting FAs, both hormone-sensitive and hormone-resistant pathways need to be blocked. Based on the differential expression of resistance pathway markers (ERBB2, BCL2, CCND1) in each human FA-derived tamoxifen-resistant organoid, we performed drug sensitivity screening by combining tamoxifen with the CDK4/6 inhibitor palbociclib, the BCL2 inhibitor venetoclax or the ERBB2 inhibitor lapatinib at the concentration of IC10 (Fig. 4h–j, Supplementary Fig. 17). Tamoxifen-resistant organoids derived from patient F111T with high ERBB2 expression were more sensitive to lapatinib and tamoxifen (Fig. 4i, Supplementary Fig. 13b), while organoids derived from patient F70T with high CCND1 and BCL2 expression had a better response to palbociclib or venetoclax plus tamoxifen (Fig. 4j, Supplementary Fig. 13b). To confirm whether there is a synergistic relationship between tamoxifen and these inhibitors, we tested the combined effect of the tamoxifen plus inhibitor with the Bliss independence model on the organoids derived from patient with high ERBB2 and CCND1 expression (Supplementary Fig. 18a). The ERBB2 inhibitor lapatinib and the CDK4/6 inhibitor palbociclib showed the strongest synergy with tamoxifen with a mean Bliss score of 17.12 and 10.21, respectively (Supplementary Fig. 18b, d). The BCL2 inhibitor venetoclax showed no synergy with tamoxifen (Bliss score = 5.14) (Supplementary Fig. 18c).

Together, these data indicated that individualized combinations of tamoxifen with CCND1, BCL2, or ERBB2 inhibitors significantly suppressed the viability of tamoxifen-resistant FAs, providing potential therapeutics for FA intervention.

## Discussion

We generated a single-cell transcriptome of human breast FAs and normal tissues to reveal the epithelial cell composition and structural characteristics of FAs, which are distinct from those of the normal breast. Previous studies have suggested that fibroadenomas are biphasic neoplasms, but the cell composition of fibroadenomas is still unclear^20^. Our scRNA sequencing analysis showed that there were 9 cell types in fibroadenoma, including mature luminal cells, luminal progenitor cells, basal cells, fibroblasts, endothelial cells, pericytes, T cells, B cells, and myeloid cells. We demonstrated that fibroblasts were enriched in stromal cells of FAs, and further analysis verified that the increase in fibroblasts seems to be not induced by higher proliferation. This result was in line with a previous study showing low KI67 expression in stromal cells in fibroadenoma^21^. To understand the relationship between epithelial cells and fibroblasts, we set up an epithelial and fibroblast coculture system and demonstrated that fibroblasts could be recruited by the epithelium of FAs. These data highlighted the important role of the epithelium in the formation of fibroadenoma.

Previous study showed that mammary glands consist of a normal bicell layer with inner luminal cells and an intact outer layer of basal cells^22^. However, the functional changes in the epithelium in FAs were ignored. Our study confirmed an increased number of luminal cells (CK8+) and a decrease in basal cells (CK14+) in breast FAs, which unexpectedly caused a loss of normal bicell structural features. Furthermore, the structural and cell composition changes in the epithelium in FAs led to estrogen-responsive functional signatures.

Our data provide key insights into the pathway of epithelial cells in FAs. We verified that, in addition to hormone-sensitive pathways, endocrine resistance pathways were activated in human FAs. This may partially explain why the therapeutic efficacy of the single estrogen receptor modulator tamoxifen has not been ideal in many prospective clinical trials^4^. A study of 58,322 fibroadenoma patients with 25.3 years of follow-up proved that biopsy-confirmed fibroadenomas are a risk factor for breast cancer^23^. Surprisingly, our data found that endocrine resistance pathways, which could be divided into 3 functional categories, ERBB2, BCL2, and CCND1, were upregulated in FA-derived breast cancer compared to FA component. These findings may provide a strategy to identify women with FA who are at high risk of developing breast cancer. However, our data were based on a small population and short follow-up time. More large retrospective studies are warranted to rigorously investigate the role of endocrine resistance markers (ERBB2, BCL2, and CCND1) in screening FA patients with a high recurrence risk and increased risk of breast cancer.

To date, there is no cell line or mouse model to study FAs. We developed the human expandable FA organoids that faithfully recapitulate the epithelium architecture and function in patients. Using this platform, we found that most patient-derived organoids were resistant to tamoxifen. Individualized combinations of tamoxifen with CCND1, BCL2, or ERBB2 inhibitors were found to significantly suppress the viability of tamoxifen-resistant organoids. However, these promising treatment options were still need to be verified in clinical trials.

In summary, this single-cell transcriptome atlas of human breast FAs and normal tissue may accelerate the development of personalized therapeutics, especially for patients with a high recurrence risk and increased risk of breast cancer.

## Methods

### Human breast fibroadenoma and normal tissue collection

Human fibroadenoma and adjacent normal breast tissues, confirmed by a pathologist, were collected from the Second Affiliated Hospital of Zhejiang University, School of Medicine. The collection of removed tissues was approved by the ethics review committee of the Second Affiliated Hospital of Zhejiang University School of Medicine (I2021001303), and informed consent for collection and research was obtained from each patient before surgery. Tamoxifen-resistant fibroadenoma was collected from patients received more than 1 year of tamoxifen treatment and experienced no size change in fibroadenoma. Each surgical specimen was obtained in rigorous sterility and minced mechanically into 1–3 mm^3^ pieces. Two random pieces were separately fixed in formalin and liquid nitrogen, while others were processed for the isolation of viable cells for further experiments. The clinical characteristics of each patient are available in Supplementary Data 1.

### Tissue processing

Tissues were cut thoroughly by scissors and plated in advanced DMEM/F12 (Sigma Life Science, 12634010) medium containing 1 mg/mL collagenase (Sigma-Aldrich, Cat.# C9407) in gentleMACS c-tubes (Miltenyi Biotec, 130-096-334). Samples were dissociated completely by a gentleMACS octo Dissociator with Heaters (Miltenyi Biotec, Germany) for 1 h under the 37C-h-TDK-3 protocol. The digested tissue suspension was strained over a 100 μm filter and pelleted at 400 × *g* for 10 min. Then, erythrocytes were lysed in 2 mL red blood cell lysis buffer (BOSTER, AR1118) for 5 min at room temperature.

### Single-cell RNA-seq using 10x Genomics Chromium

A 10x Genomics Single Cell 3’ v3 kit was used to capture single cells. cDNA libraries were obtained from fibroadenoma and normal breast tissues according to the manufacturer’s instructions and then pooled and sequenced by NovaSeq 6000.

### Single-cell RNA-seq quality control

Sequenced reads obtained from NovaSeq 6000 were demultiplexed and mapped to the GRCh38 human reference genome. Downstream single-cell analyses were performed using the Cell Ranger (version 4.0.0, 10x Genomics) and Seurat R packages (V 3.2.0). Unique molecule identifiers (UMIs) were counted to generate digital expression matrices for each gene, and each cell barcode was filtered by CellRanger. We applied secondary filtration by Seurat to discard genes and cells according to the following criteria: (1) genes expressed in <3 cells; (2) cells with <200 or >5000 expressed genes; (3) cells that contained <500 UMIs; and (4) mitochondrial read ratio >25%. Samples were then integrated and normalized.

### tSNE analysis and cell cluster identification of total cells in single-cell RNA-seq

This study included 28,101 cells, with 17,776 from fibroadenoma (F8T: 5297, F51T: 4389, F131T: 3980, F132T: 4110) and 10,325 from normal tissue (F8N: 3194, F51N: 7131). We identified 2000 variable genes for the clustering of all cell types and performed t-distributed stochastic neighbor embedding clustering (t-SNE) using the Seurat R Package with the 30 most informative principal components (PCA). Cell types were annotated by a combination of marker genes identified from the literature^13^. This analysis identified 10 clusters of cells: mature luminal cells (MLs, expressing *EPCAM*, *KRT8*, *KRT18*, *CITED1*, *ESR1*, and *PGR*), luminal progenitor cells (LPs, expressing *EPCAM*, *KRT8*, *KRT18*, *KRT15*, *CD24*, and *ELF5*), basal cells (BCs, expressing *KRT5*, *KRT14*, and *KRT17*), endothelial cells (EC, expressing *PECAM*, *CDH5*, and *ENG*), fibroblasts (FBs, expressing *PDGFRA*), pericytes (PTs, expressing *RGS5*), T cells (T, expressing *PTPRC*, *CD3D*, and *CD3E*), B cells (B, expressing *PTPRC*, *CD79A*, and *CD79B*), myeloid cells (M, expressing *PTPRC*, *LYZ*, and *C1QB*) and an unknown cell population (Un). We compared the distributions of different cell subtypes in fibroadenomas and adjacent normal tissues.

### tSNE analysis and cell cluster identification of epithelial cells in single-cell RNA-seq

Hierarchical clustering of epithelial cell communities was performed on the basis of classic marker genes, including luminal lineage (expressing *EPCAM*, *KRT8*, *KRT18*) and myoepithelial lineage (expressing *KRT5*, *KRT14*, *KRT17*). In addition, we integrated four single-cell datasets of normal human mammary epithelial cells downloaded from the NCBI database GSE113197 due to the difficulty in obtaining enough paired normal tissues for single-cell RNA sequencing. This study eventually included 6346 epithelial cells, split evenly between fibroadenoma (F8T: 276, F51T: 1592, F131T: 1014, F132T: 291) and normal cells (F8N: 138, F51N: 173, Ind4: 485, Ind5: 830, Ind6: 710, Ind7: 837). We subsequently performed t-SNE to identify cell clusters and compared cell contributions between fibroadenoma and normal tissues.

### Differentially expressed genes in single-cell RNA-seq

Differentially expressed gene (DEG) analysis was conducted between fibroadenoma and normal epithelial cells using the FindMarkers and FindAllMarkers functions of the Seurat R package. The Wilcoxon rank-sum test (two-sided) was employed to identify DEGs between the two groups of epithelial cells. The log-transformed fold change threshold was set to 0.50. The parameter ‘min.pct’ was set to 0.10.

### Pathway analysis in single-cell RNA-seq

GO and KEGG pathway analyses were performed using the enrichGO and enrichKEGG functions of the clusterProfiler (V 3.18.0) R package. GSEA was used based on the KEGG pathway by the gseKEGG function. Gene sets or pathways were determined by a hypergeometric test (one-sided), and adjusted *p* < 0.05 or FDR *q* < 0.25 was considered statistically significant.

### Violin plots for gene expression in single-cell RNA-seq

Violin plots were plotted for expression values of the indicated genes using the VlnPlot function of the Seurat R package. The two-sided Wilcoxon rank-sum test was used to evaluate the statistical significance (****p < 0.0001) of differences between groups by the ggplot2 (V 3.3.2) and ggpubr (V 0.4.0) packages.

### Flow cytometry analysis

The dissociated single cells from primary fibroadenoma tissues or normal tissues were resuspended in 100 μl of cell staining buffer (Biolegend, Cat.# 420201) and stained with Zombie Red™ (Biolegend, Cat.# 423109) to distinguish live cells for 30 min at room temperature according to the manufacturer’s instructions. Then, the cells were pelleted at 400 × *g* for 10 min at 4 °C, resuspended in 100 μl of cell staining buffer and stained with a series of FACS antibodies, including CD45 (Biolegend, clone: HI30, cat. # 304014, [1:100]), CD31 (Biolegend, clone: WM59, cat. # 303118, [1:100]), EpCAM (Biolegend, clone: 9C4, cat. # 324204, [1:100]) and CD49f (Biolegend, clone: GoH3, cat. # 313612, [1:100]). Staining was performed for 30 min with FACS antibodies on ice and washed twice with processing buffer (PBS, 5% BSA). After washing, the cells were resuspended in 200–300 μL of cell staining buffer and analyzed on a FACSCanto II flow cytometer (BD Biosciences). Cell sorting was performed on a FACSAria III cell sorter (BD Biosciences). Data were analyzed with FlowJo software (V10.0 for Windows).

### Immunostaining of fibroadenoma and normal breast tissue sections

Tissues were fixed in 4% paraformaldehyde (PFA) for 24 h, subsequently embedded in paraffin, and sectioned into 10 µm sections that were mounted on glass slides. Sections were stained with standard hematoxylin and eosin (HE) immunohistochemical staining.

To perform immunofluorescent staining, sections were blocked with endogenous catalase blocker for 15 min and 3% BSA (MP Biomedicals, Cat.# 0218054990) for 40 min. Then, sections were incubated with primary antibody at 4 °C overnight. The primary antibodies were as follows: ER (Leica, clone: 6F11, Cat.# PA0151, [1:600]), PR (Leica, clone: 16, Cat.# PA0312, [1:600]), ERBB2 (Roche, clone: 4B5, Cat.# 05999570001, [1:1]), BCL2 (ZSGB-BIO, clone: EP36, Cat.# ZA-0536, [1:400]), CCND1 (ZSGB-BIO, clone: SA38-08, Cat.# ZA-0101, [1:800]), TFF3 (Abcam, clone: EPR3974, Cat.# ab108599, [1:500]), CK8 (Hangzhou HuaAn Biotechnology, colon: A1-B11, Cat.# M1603-2, [1:100]), CK14 (Abcam, colon: EP1612Y, Cat.# ab51054, [1:100]), PRLR (Abcam, clone: EPR7184(2), Cat.# ab170935, [1:100], TFF1 (Abcam, Clone: EPR3972, Cat.#ab92377, [1:100]), KIAA1324 (Novus, Cat.#NBP2-57699, [1:500]). Sections were washed with PBST (PBS with 0.1% Tween) three times and stained with secondary antibody for immunohistochemical staining (IHC) (horseradish peroxidase-conjugated anti-mouse&rabbit: absin, Cat.# 996, [1:200]) or immunofluorescence staining (IF) (Goat pABs to Rb IgG (Alexa Fluor® 488): Abcam, Cat.# ab150081; Goat pABs to Ms IgG (Alexa Fluor® 555): Abcam, Cat.# ab150118, [1:200]) for 2 h at room temperature. Sections were washed with PBST three times and stained with hematoxylin for IHC or DAPI (Invitrogen, Cat.# D1306) for IF. Sections were visualized with a laser scanning confocal fluorescence microscope (LSM800, Carl Zeiss, Germany) or digital slice scanner (VS200, Olympus, Japan). The analyses of immunostaining were performed with imageJ2, ZEN 3.4 (blue edition), and Olympus Image Viewer 3.

### IHC score

The IHC score was calculated with the following equation: H-score = (1 × % of positive cells with weak staining) + (2 × % of positive cells with moderate staining) + (3 × % of positive cells with strong staining). Two-sided Fisher’s exact tests (H-score as categorical data) were performed to evaluate differences in relapse rate between groups of high/low ERBB2, BCL2, and CCND1 protein expression, as shown in Table 1. ERBB2 (two-sided Fisher’s exact tests), BCL2, and CCND1 (unpaired two-tailed Student’s t test) protein expression was analyzed between relapsed fibroadenoma tissues and non-relapsed tumors, as shown in Fig. 3d, and between fibroadenoma and cancer derived from fibroadenoma, as shown in Fig. 3e.

### Organoid culture

The pellet from tissue processing was resuspended in cold Matrigel matrix (BD, 356231) and seeded on a 24-well plate at 37 °C for 15 min. Then, 400 μl organoid culture medium (Advanced DMEM/F12 (Gibco, 12634-010) supplemented with GlutaMAXTM-I (Gibco, 35050-061), B27 (Gibco, 17504-044), SB202190 (Sigma-Aldrich, Cat.# S7067), EGF (Peprotech, Cat.# AF-100-15), Neuregulin 1 (Peprotech, Cat.# 100-03), Noggin (Peprotech, Cat.# 120-10C), R-spondin1 (Peprotech, Cat.# 120-38), A-83-01 (tocris, Cat.# 2939), Y27632 (abmole, Cat.# Y-27632), KGF (Peprotech, Cat.# 100-19), FGF10 (Peprotech, Cat.# AF-100-26), N-acetylcysteine (Sigma, Cat# A9165-5 g), nicotinamide (Sigma, Cat# N0636), β-estradiol (Sigma, Cat# E2758), primocin (Life Technologies, ant-pm-1) and penicillin/streptomycin (Life Technologies, 1:100)) was added. Organoids were incubated in 37 °C, 5% CO_2_, humid incubators (Thermo Fisher). The medium was changed every 3 days. Organoids were passaged at ratios (1:1 to 1:4) for 1–4 weeks using TryPLETM Express (Gibco, 12605-028), digested for 5 min, and centrifuged at 400 × *g* for 10 min. Organoids were frozen using serum-free cell saving solution (Biotech, Cat.# C40100) in liquid nitrogen. Most of the fibroadenoma tissues received were sufficient for different types of experiments. Organoids at the same passage were used for drug-sensitive test, whole-exome sequencing, RNA sequencing, and immunostaining.

### Limited RNA-seq

Total RNA of epithelial cells sorted by fluorescence-activated cell sorting was extracted using TRIzol reagent (Ambion, 15596018). Then, total RNA was qualified and quantified using a NanoDrop and Agilent 2100 bioanalyzer (Thermo Fisher Scientific, MA, USA). Limited RNA was amplified and reverse transcribed to cDNA. Purified cDNA was fragmented into small pieces and transferred to single-strand circular DNA (ssCir DNA), which was formatted as the final library. The final library was amplified with phi29 (Thermo Fisher Scientific, MA, USA) to make DNA nanoballs (DNBs) with more than 300 copies of a molecule. DNBs were loaded into the patterned nanoarray, and paired-end 100-base reads were constructed on the MGISEQ-2000 platform (BGI-Shenzhen, China).

### Limited RNA-seq quality control

The final library was quantitated by an Agilent 2100 bioanalyzer instrument to determine the average molecule length, and real-time quantitative PCR (qPCR) was used to quantify the library. The sequencing data were filtered with SOAPnuke (v1.5.2). Filtering strategies were applied to remove reads (1) that contained sequencing adapters; (2) whose low-quality base ratio (base quality ≤5) was >20%; and (3) whose unknown base (‘N’ base) ratio was >5%. The clean reads were mapped to the GRCh38 human reference genome using HISAT2 (v2.0.4) and aligned to the reference coding gene set by Bowtie2 (v2.2.5). RSEM (v1.2.12) was applied to calculate the expression level of the gene.

### Whole-exome sequencing

Total DNA of fibroadenoma tissues (Tt), fibroadenoma organoids (To), and matched peripheral blood mononuclear cells (PBMCs) were extracted using a QIAwave DNA Blood & Tissue Kit (Cat. No. 69556). Total DNA was qualified and quantified using a NanoDrop and Agilent 2100 bioanalyzer (Thermo Fisher Scientific, MA, USA). Genomic DNA was fragmented into small pieces, and specialized adaptors were ligated to both fragment ends, which was constructed as the library. Then, the hybridization of fragments in the library was performed, followed by bridge amplification cycles of each bound fragment. Single-stranded circular DNA molecules were amplified to DNA nanoballs, which were added to the chip using high-density DNA nanochip technology. Two-terminal sequences with a read length of 150 bp were obtained on the DNBSEQ platform. The raw sequencing data were filtered and then mapped against the human reference genome GRCh38 using Burrows-Wheeler Aligner (v0.7.17). BAM files were analyzed according to best practices workflows of the Genome Analysis Toolkit (GATK). GATK4-HaplotypeCaller (v4.1.4.1) mode was utilized to generate variant call format files (VCFs). VCF files were converted into mutation annotation format using Snpeff (5.1).

### Drug test

Organoids were harvested and diluted to 100 organoids/ml in organoid culture medium containing 5% cold Matrigel matrix. White 384-well plates (Greiner, REF: 781080) were coated with 20 μL of 50% cold Matrigel matrix using a Multidrop combi reagent dispenser (Thermo Fisher, America). After solidifying at 37 °C for 30–60 min, we added 20 μL of organoid suspension per well to the same plate. The plate was incubated at 37 °C and 5% CO_2_ overnight. Drugs were added in triplicate using a 12-channel manual pipette (Eppendorf, Germany). A CellTiter-Glo® 3D Cell Viability Assay (Promega, G9683) was used to measure cell viability, and luminescence was measured on a SpectraMax® iD3 multifunction reader (Molecular Devices, America).

Drug concentration strategies were as follows: (1) Single drug sensitivity test: tamoxifen (Selleckchem, Cat.# S1238, stock 10 mM in DMSO) with 10 concentration gradients and DMSO control were used. (2) Drug combination test, we screened the tamoxifen-resistant organoid lines by combining with the candidate drugs at a concentration of IC10 (which was obtained from the first round of drug testing). Groups were (1) DMSO control; (2) Tamoxifen (used at 10 μM); (3) Tamoxifen (10 μM) + Lapatinib (Selleckchem, Cat.# S2111, stock 10 mM in DMSO, used at 10 nM); (4) Tamoxifen (10 μM) + Venetoclax (Aladdin, Cat.# A124869, stock 10 mM in DMSO, used at 5 μM); (5) Tamoxifen (10 μM) + Palbociclib (Selleckchem, Cat.# S1579, stock 10 mM in DMSO, used at 10 nM). The results are presented as the mean with SEM, and *p* values were calculated using one-way ANOVA (*p < 0.05, **p < 0.01, ***p < 0.001, and ****p < 0.0001).

### Statistics

Data are presented as the mean ± SEM. The unpaired two-tailed Student’s t test was used in flow cytometry analysis and immunostaining quantification. One-way ANOVA was used in drug combination test analysis. ERBB2 (Fisher’s exact tests), BCL2, and CCND1 (unpaired two-tailed Student’s t test) protein expression was assessed between relapsed fibroadenoma tissues and non-relapsed tumors, as shown in Fig. 3d, and between fibroadenoma tissues and cancer derived from fibroadenoma, as shown in Fig. 3e.

### Reporting summary

Further information on research design is available in the Nature Portfolio Reporting Summary linked to this article.

## Supplementary information

Supplementary Information

Reporting Summary

Description of Additional Supplementary Files

Supplementary Data 1

Supplementary Data 2

Supplementary Data 3

Supplementary Data 4

Supplementary Data 5

Supplementary Data 6

Supplementary Data 7

Supplementary Data 8

Supplementary Data 9

Supplementary Data 10

Supplementary Data 11

Supplementary Data 12

Supplementary Data 13

Supplementary Data 14

## Data Availability

The raw data of Single-cell RNA sequencing, limited RNA sequencing, and whole-exome sequencing have been deposited in the Genome Sequence Archive (GSA) (https://ngdc.cncb.ac.cn/gsa/) in the National Genomics Data Center, China National Center for Bioinformation/Beijing Institute of Genomics under accession number HRA002242. Access to data will be granted following registration in the NGDC system as an approved user. The single-cell datasets of normal human mammary epithelial cells used in this study are available in the NCBI database under accession code GSE113197. The remaining data generated in this study are provided in the Supplementary Information/Source data file. Source data are provided with this paper.

## Source data

Source Data

## Code availability

Custom codes were not generated for data analyses in this study. Publicly available software and algorithms were used to perform data analyses.
