# Supplementary material for: Single cell profiling of female breast fibroadenoma reveals distinct epithelial cell compositions and therapeutic targets
Source: Nat Commun. 2023 Jun 16;14:3469. doi: 10.1038/s41467-023-39059-3 (PMC10275980; doi:10.1038/s41467-023-39059-3)
Supplement: Supplementary file 1 — Supplementary Information [file 41467_2023_39059_MOESM1_ESM.pdf]

Single-cell profiling of female breast fibroadenoma reveals distinct epithelial cell compositions and therapeutic targets

Zhigang Chen[1,2,3,7], Yi Zhang[1,2,3,4,7], Wenlu Li[5,7], Chenyi Gao[1,2,3,4], Fengbo Huang[6], Lu Cheng[6], Menglei Jin[1,2,3], Xiaoming Xu[6], Jian Huang[1,2,3,4]

[1]Department of Breast Surgery (Surgical Oncology), Second Affiliated Hospital, Zhejiang University School of Medicine, 88 Jiefang Road, Hangzhou, Zhejiang, China
[2]Key Laboratory of Tumor Microenvironment and Immune Therapy of Zhejiang Province, Hangzhou, China
[3]Cancer Centre, Zhejiang University, Hangzhou, Zhejiang, China
[4]Cancer Institute, Key Laboratory of Cancer Prevention and Intervention, Ministry of Education, The Second Affiliated Hospital, Zhejiang University School of Medicine, Hangzhou, Zhejiang, China
[5]Departments of Radiology, Massachusetts General Hospital, Harvard Medical School, Boston, MA 02129, USA
[6]Department of Pathology, The Second Affiliated Hospital, Zhejiang University School of Medicine, Hangzhou, Zhejiang, China
[7]These authors contributed equally: Zhigang Chen, Yi Zhang, Wenlu Li
e-mail: drhuangjian@zju.edu.cn; chenzhigang@zju.edu.cn

METHODS

In vitro coculture system

Primary fibroblasts and epithelium were isolated from human fibroadenoma and adjacent normal breast tissues as described in previous studies [1,2.] Briefly, the digested tissue was placed in 50 mL sterile tubes and centrifuged at 40 g for 1 min, and the organoid fraction was obtained. The supernatant was transferred into new 50 mL tubes and centrifuged at 100 g for 2 min to obtain the pellet as the epithelial fraction. The rest of the sample was centrifuged at 200 g for 4 min to obtain the stromal fraction. The supernatant enriched with stromal cells was moved to new tubes and centrifuged at 250 g for 10 min. The pellet was harvested and cultured in DMEM with 10% FBS. Fibroblasts were passaged within 5 population doublings after isolation. The purity of epithelial and fibroblast cells was identified by flow cytometry. Isolated fibroblasts and epithelial cells were suspended in Advanced DMEM/F12 medium containing 10% FBS, and a coculture assay was performed.

1) Fibroblasts from normal breast tissues suspended in medium at $2X10^5$/mL. Fibroblasts (200 μL) were placed in the upper compartment of a Transwell chamber with 8 μm pores (Corning). Isolated epithelial cells from normal and fibroadenoma tissues were then added to the bottom chamber at a concentration of 100,000 cells/well in 600 μl medium. After incubation for 12 h at 37°C and 5% CO2, the Transwell chamber was removed and washed with PBS. 2) 200 μL epithelial cells from normal breast tissues were inoculated into the upper chamber of a Transwell plate at $2X10^5$/mL. Fibroblasts from normal and fibroadenoma tissues were plated into the bottom chamber at a concentration of 100,000/well in 600 μl medium. After incubation for 48 h at 37°C and 5% CO2, the Transwell chamber was removed and washed with PBS. The transwell

aperture was fixed in 4% paraformaldehyde for 15 min and sequentially stained with crystal violet for 20 min. The nonmigrating cells in the upper chamber were removed and observed under a microscope.

Organoid embedding for immunostaining

Organoids were dyed with 0.05% crystal violet dye for 15-30 min and fixed in 4% PFA for 24 h. Then, organoids with Matrigel were scraped off using a flat pipette tip that could be cut off, transferred to 2% semisolidified agarose, and subsequently wrapped in liquid agarose.

Real-time quantitative PCR

Total RNA was extracted using TRIzol Reagent (Invitrogen) and reverse-transcribed into cDNA using Hifair® V one-step RT-gDNA digestion SuperMix for qPCR (Yeasen, Cat.# 11142ES10). Real-time quantitative PCR was performed using a Hifair® Ⅲ One Step RT–qPCR SYBR Green Kit (Yeasen, Cat.# 11143ES50) with an Applied Biosystem 7500 instrument (Applied Biosystems). Primer sequences of genes related to the estrogen response are listed in Supplementary Data 11. The mRNA expression of genes relative to β-actin was calculated using the ΔΔCt method. The results are presented as the mean with SEM, and p values were calculated using paired Student's t test and one-way ANOVA (*p <0.05, **p <0.01, ***p <0.001, and ****p <0.0001).

Bliss independence model for synergy score

We produced the dose-response matrix data using cell viability assays as described in the drug test.Tamoxifen (18, 10, 5.6, 3.2, 1, 0.1, 0μM) was tested in combination with lapatinib (1.2, 0.275, 0.1, 0.036, 0.012, 0μM), venetoclax (36,

10, 3.6, 1.2, 0.1, 0μM) and palbociclib (3.6, 1.2, 0.275, 0.1, 0.012, 0μM). Bliss synergy score was used to calculate synergy for each drug combination by SynergyFinder web application (version 3.0) https://synergyfinder.fimm.fi/synergy/20230329123549337715/).

Statistics

Data are presented as the mean ± SEM. The unpaired two-tailed Student's t test was used in immunostaining quantification. Fisher's exact tests (H-score as categorical data) were performed to evaluate differences in relapse rate between groups of high/low ERBB2, BCL2 and CCND1 protein expression.

SUPPLEMENTARY REFERENCES

1 Orimo, A. *et al.* Stromal fibroblasts present in invasive human breast carcinomas promote tumor growth and angiogenesis through elevated SDF-1/CXCL12 secretion. *Cell* **121**, 335-348, doi:10.1016/j.cell.2005.02.034 (2005).

2 Zubeldia-Plazaola, A. *et al.* Comparison of methods for the isolation of human breast epithelial and myoepithelial cells. *Front Cell Dev Biol* **3**, 32, doi:10.3389/fcell.2015.00032 (2015).

Supplementary Information

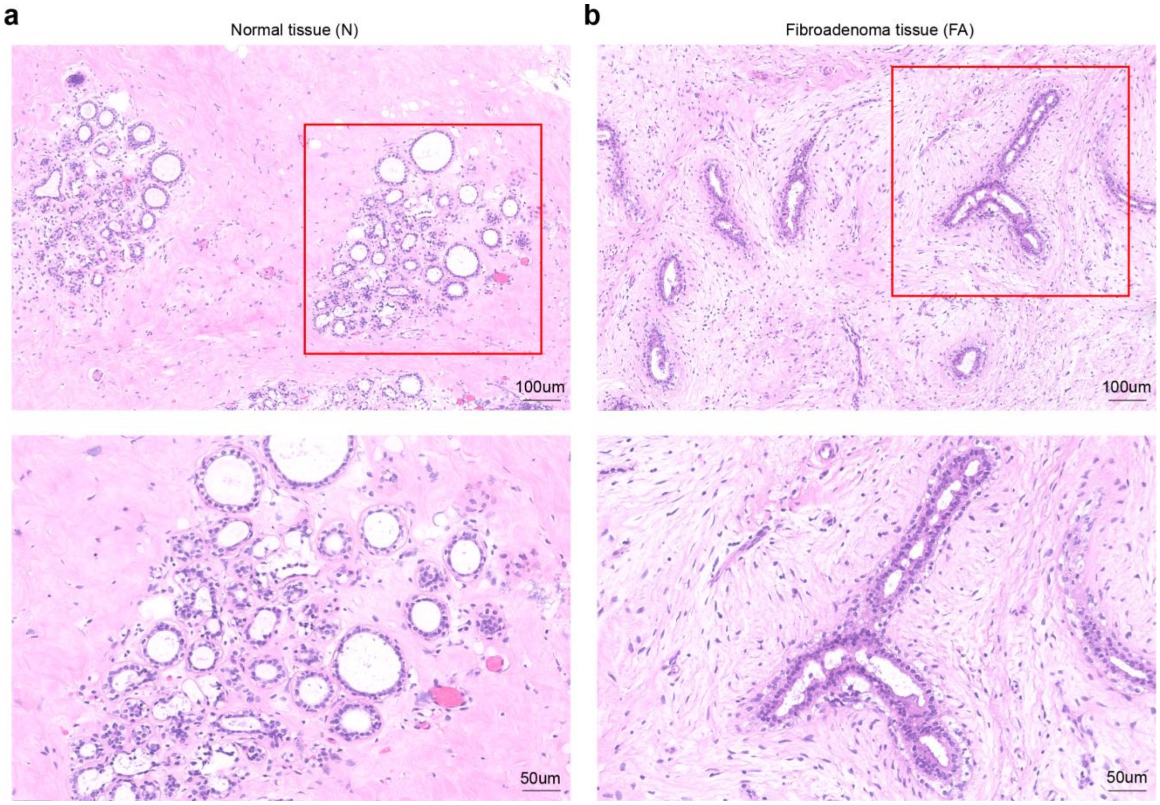

Supplementary Figure 1. Histopathological diagnosis of fibroadenoma and normal tissue

a) HE staining of normal tissue. Experiment was performed with three independent replicates.

b) HE staining of fibroadenoma shows a typical appearance of FAs with hyalinized stroma around epithelial elements that are stretched into arc-like formations. Scale bars: 100 μm and 50 μm. Experiment was performed with three independent replicates.

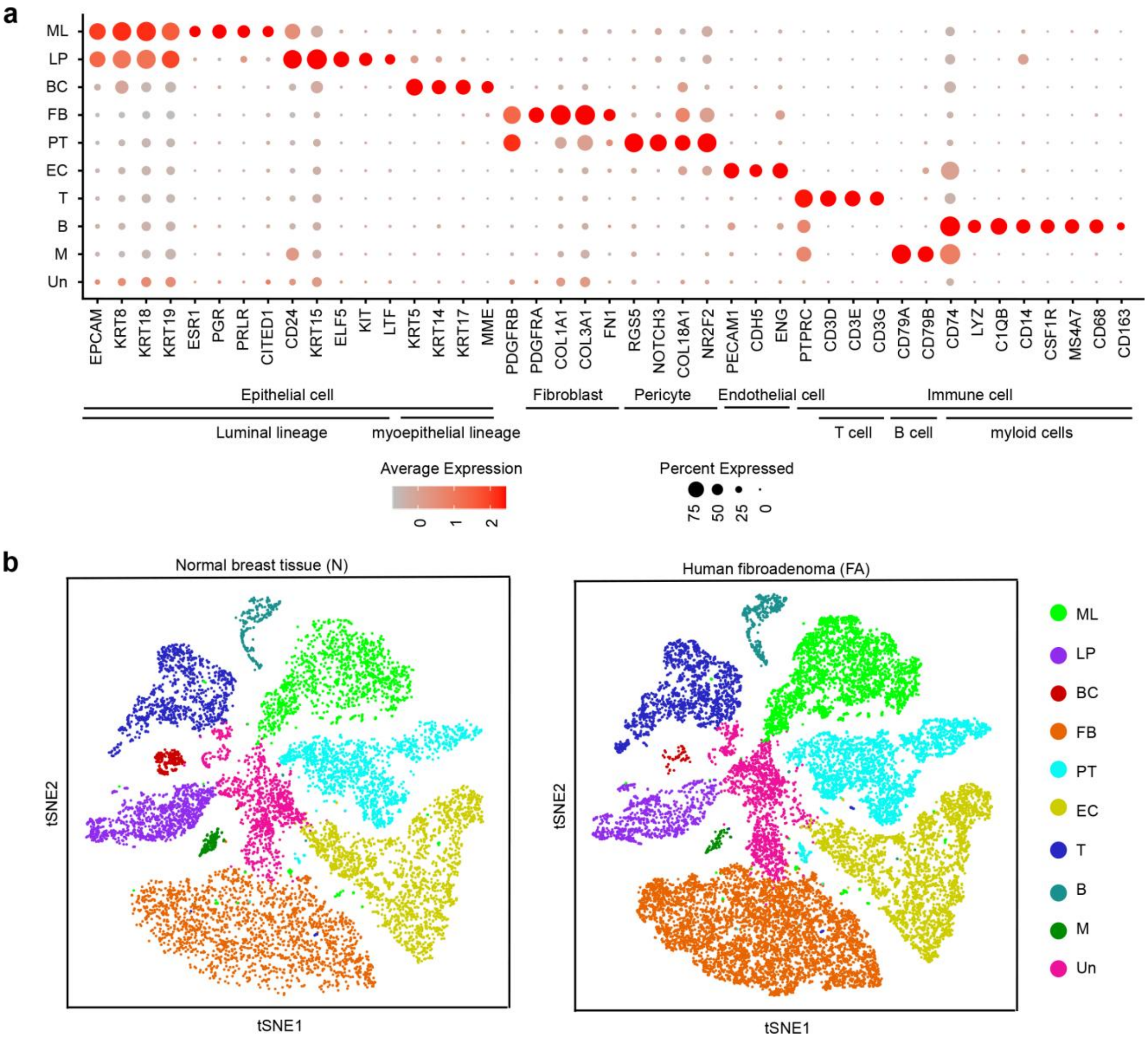

Supplementary Figure 2. Cell composition in fibroadenoma and normal tissue

a) Expression of canonical markers of epithelial, fibroblast, pericyte, endothelial and immune cells.

b) t-SNE clustering of 28,101 single-cell transcriptomes colored by significant cell type clusters and split by group.10 cell clusters are shown as mature luminal cells (ML), luminal progenitor cells (LP) and basal cells (BC), fibroblasts (FB), endothelial cells (EC), pericytes (PT), T cells (T), B cells (B), myeloid cells (M) and unknown cells (Un).

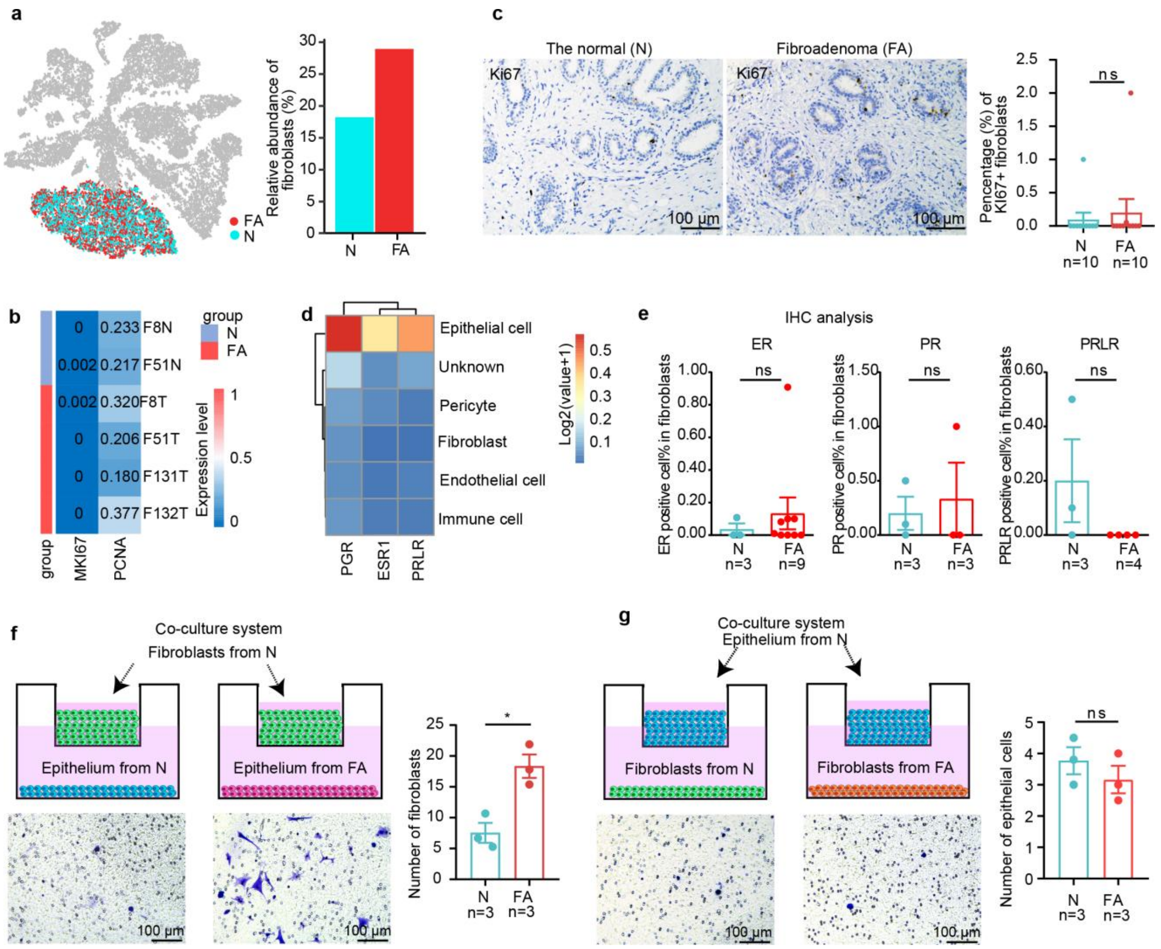

Supplementary Figure 3. The epithelial cells in fibroadenoma recruit fibroblasts from normal tissue

a) The tsne plot (left) and bar plot (right) show that the relative abundance of fibroblasts in fibroadenoma tissues was increased compared to that in normal tissues.

b)Heatmap of the expression of proliferation-associated genes (*MKI67, PCNA*) in fibroadenoma(FA) and normal tissues(N).

c)Immunohistochemical staining (left) and the scatter diagram (right) show the percentages of KI67-positive cells in fibroblasts between fibroadenoma and normal tissues. Scale bar: 100 µm. Data are expressed as the mean +/− s.e.m. Each dot represents one patient, ns p=0.1837 (paired two-tailed Student’s t test).

d) Heatmap of the expression of hormone receptors (*PGR*, *PRLR*, *ESR1*) in epithelial, fibroblast, pericyte, endothelial and immune cells.

e) The scatter diagram shows the percentages of PGR+, PRLR+, and ESR1+ cells in fibroblasts between fibroadenoma and normal tissues. Data are expressed as the mean +/− s.e.m. Each dot represents one patient, ER ns p=0.59; PR ns p=0.73; PRLR ns p=0.18 (unpaired two-tailed Student's t test).

f) Coculture system. Crystal violet staining (left) and the scatter diagram (right) show the counts of normal fibroblasts migrating to the epithelial cells from the normal (N) and fibroadenoma (FA) for 12 h. Scale bar: 100 μm. n = 3/group from three independent experiments. Data are expressed as the mean +/− s.e.m. *p=0.0178 (unpaired two-tailed Student's t test).

g) Crystal violet staining (left) and the scatter diagram (right) show the counts of normal epithelial cells migrating to the fibroblasts from the normal (N) and fibroadenoma (FA) for 48 h. Scale bar: 100 μm. n = 3/group from three independent experiments. Data are expressed as the mean +/− s.e.m. ns p=0.4499 (unpaired two-tailed Student's t test).Source data are provided as a Source Data file.

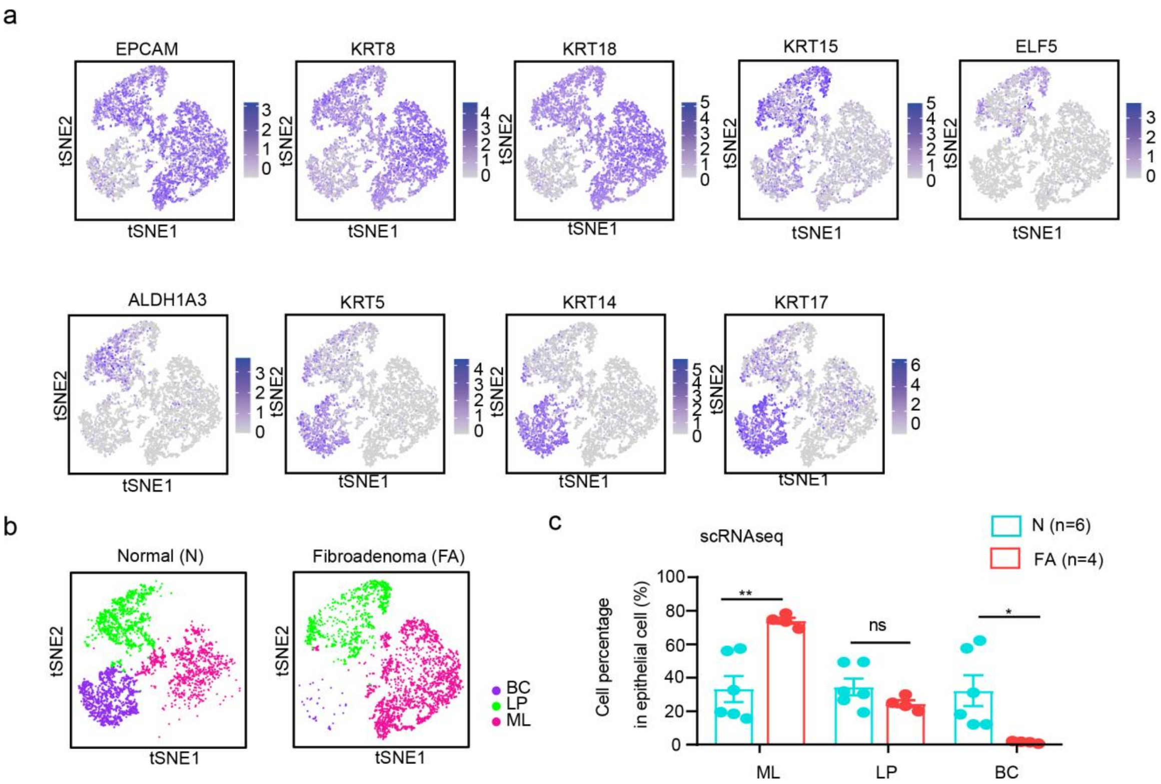

Supplementary Figure 4. The cell composition of epithelial cells in fibroadenoma and normal tissue

a) Canonical markers of the epithelial cell subset.

b) t-SNE clustering of 6,346 single-cell transcriptomes colored by significant cell type clusters and split by group (3,173 from fibroadenoma and 3,173 from normal tissues).

c) Distribution of epithelial subsets. Data are expressed as the mean +/− s.e.m. Each dot represents one patient. **p=0.0033 (ML), ns p=0.1636 (LP), *p=0.0286 (BC), unpaired two-tailed Student's t test. Source data are provided as a Source Data file.

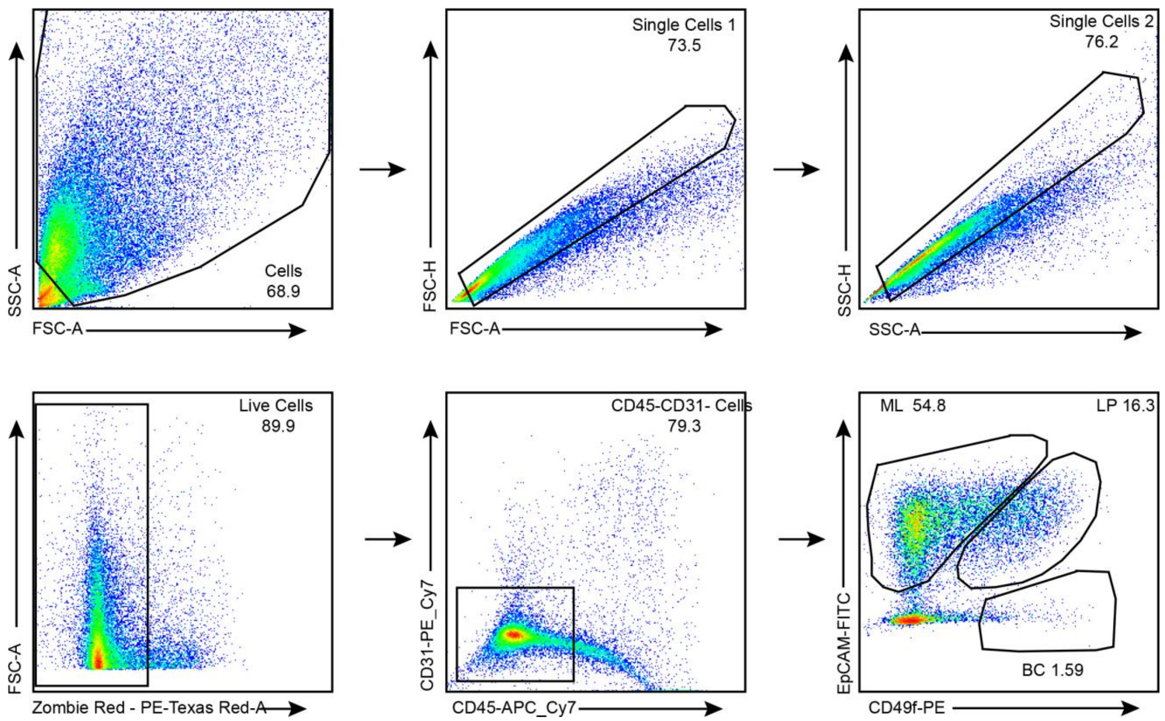

Supplementary Figure 5. FACS gating/sorting strategy. FACS scheme for the isolation of CD45−CD31− cells from the fibroadenoma for Figure 1g, Figure 4f, Supplementary Figure 11b and sample preparation of Figure 4d, e.

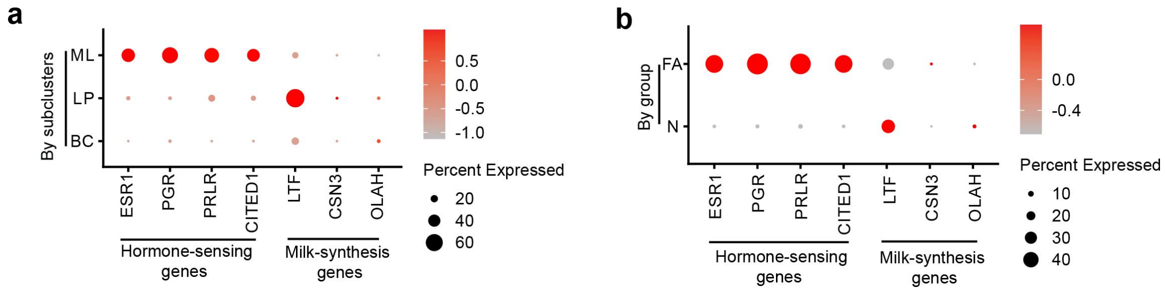

Supplementary Figure 6. Each epithelial cell subset in fibroadenoma displays a distinct functional signature

a, b) Hormone-sensing and milk synthesis gene groups by subcluster (a) and group (b).

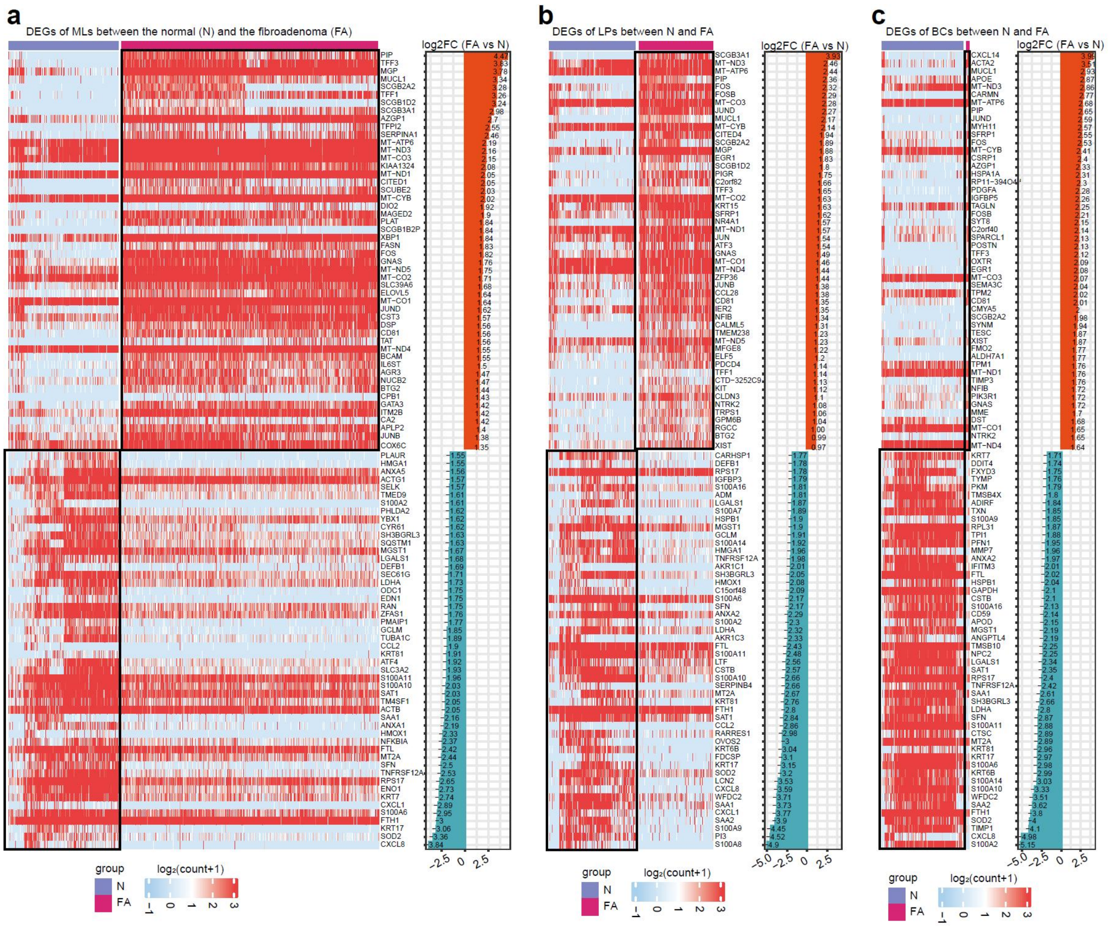

Supplementary Figure 7. DEGs of 3 different epithelial cell types, including mature luminal cells, luminal progenitor cells, and basal cells, between fibroadenomas and normal tissues

a) Heatmap showing the distribution of upregulated and downregulated top DEGs identified in mature luminal cells (MLs) between fibroadenomas and normal tissues (left); bar plot showing the log2FC of the DEGs between fibroadenomas and normal tissues (right).

b) Heatmap showing the distribution of upregulated and downregulated top DEGs identified in luminal progenitor cells (LPs) between fibroadenomas and normal tissues (left); bar plot showing the log2FC of the DEGs between fibroadenomas and normal tissues (right).

c) Heatmap showing the distribution of upregulated and downregulated top DEGs identified in basal cells (BCs) between fibroadenomas and normal tissues (left);

bar plot showing the log2FC of the DEGs between fibroadenomas and normal tissues (right).

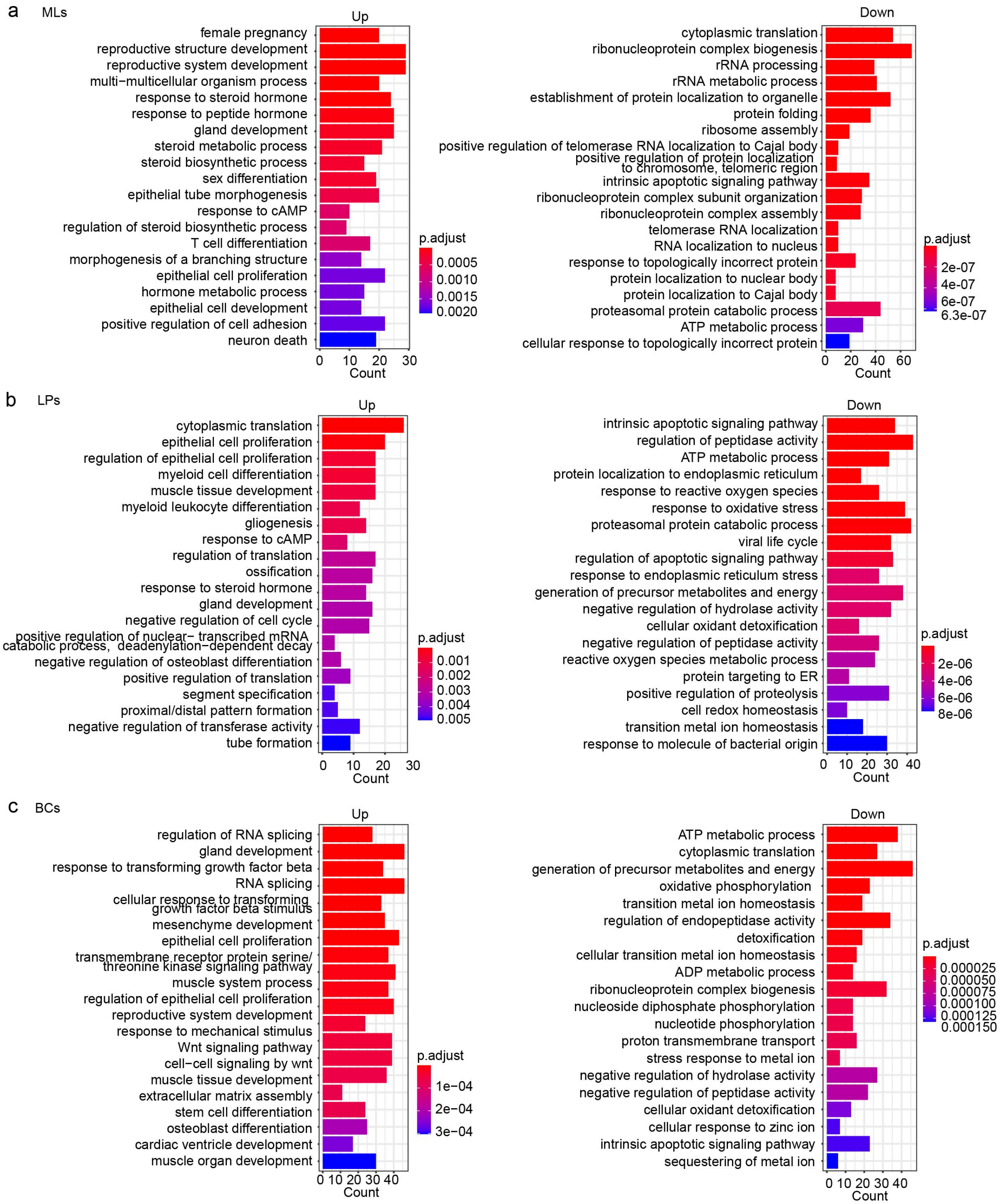

Supplementary Figure 8. GO enrichment analysis of DEGs in mature luminal cells, luminal progenitor cells, and basal cells between fibroadenomas and normal tissues

a) Representative GO terms of upregulated (left) and downregulated (right) DEGs in the mature luminal cells(MLs) between fibroadenomas and normal

tissues. One-sided, hypergeometric test and adjusted by Benjamini-Hochberg correction.

b) Representative GO terms of upregulated (left) and downregulated (right) DEGs in the luminal progenitor cells (LPs) between fibroadenomas and normal tissues. One-sided, hypergeometric test and adjusted by Benjamini-Hochberg correction.

c) Representative GO terms of upregulated (left) and downregulated (right) DEGs in the basal cells (BCs) between fibroadenomas and normal tissues.One-sided, hypergeometric test and adjusted by Benjamini-Hochberg correction.

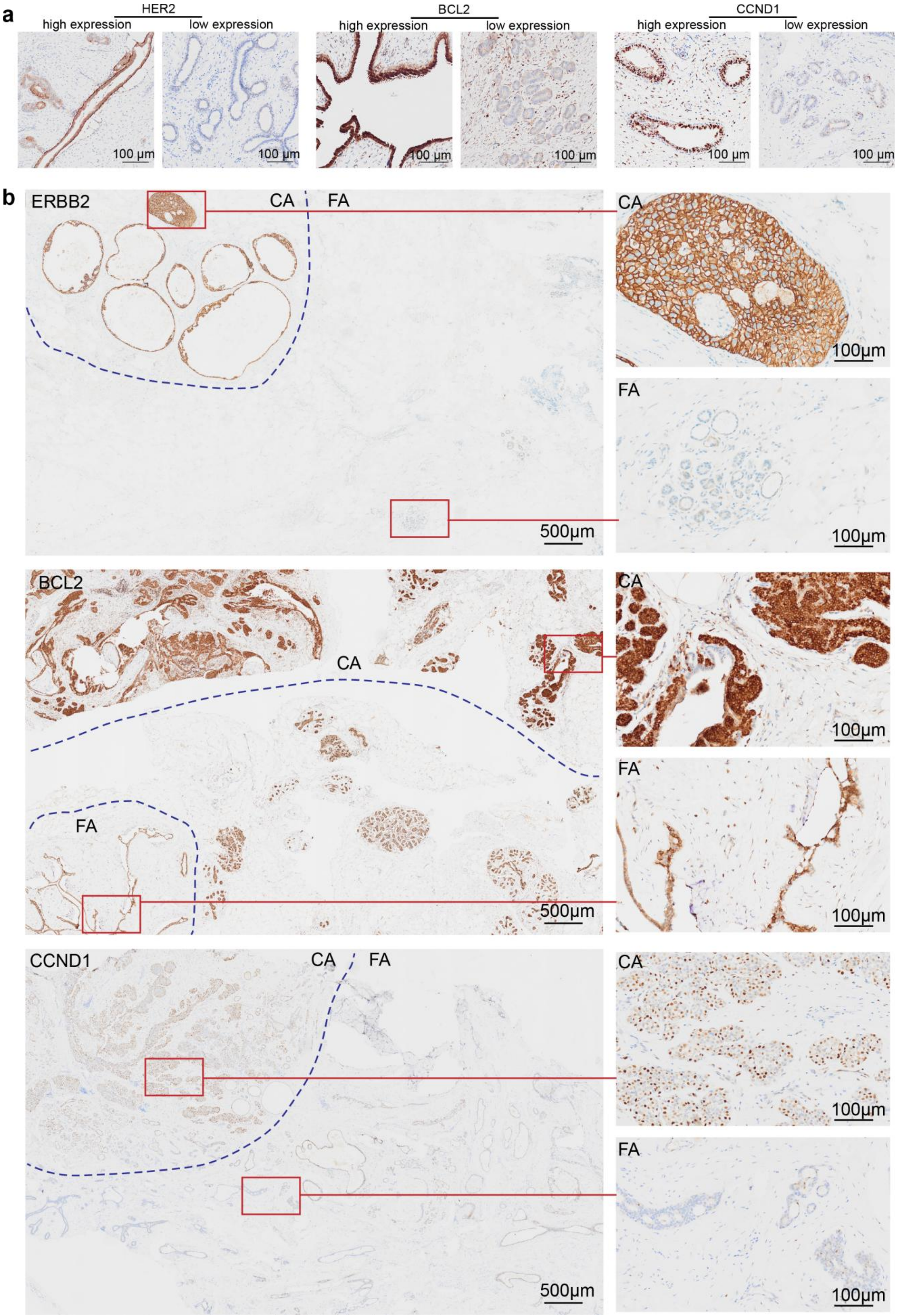
a
HER2
high expression
low expression
100 µm
100 µm
BCL2
high expression
low expression
100 µm
100 µm
CCND1
high expression
low expression
100 µm
100 µm
b
ERBB2
CA
FA
500µm
CA
100µm
FA
100µm
BCL2
CA
FA
500µm
CA
100µm
FA
100µm
CCND1
CA
FA
500µm
CA
100µm
FA
100µm

Supplementary Figure 9. Representative IHC staining of endocrine resistance markers

a) Representative IHC staining of endocrine resistance markers (ERBB2, BCL2, CCND1) at high and low levels. Scale bar: 100 μm. Experiment was performed with three independent replicates.

b) Representative IHC staining of endocrine resistance markers (ERBB2, BCL2, CCND1) in 8 patients with breast cancer who had been pathologically proven to be derived from FAs was analyzed. Scale bars: 500 μm and 100 μm.

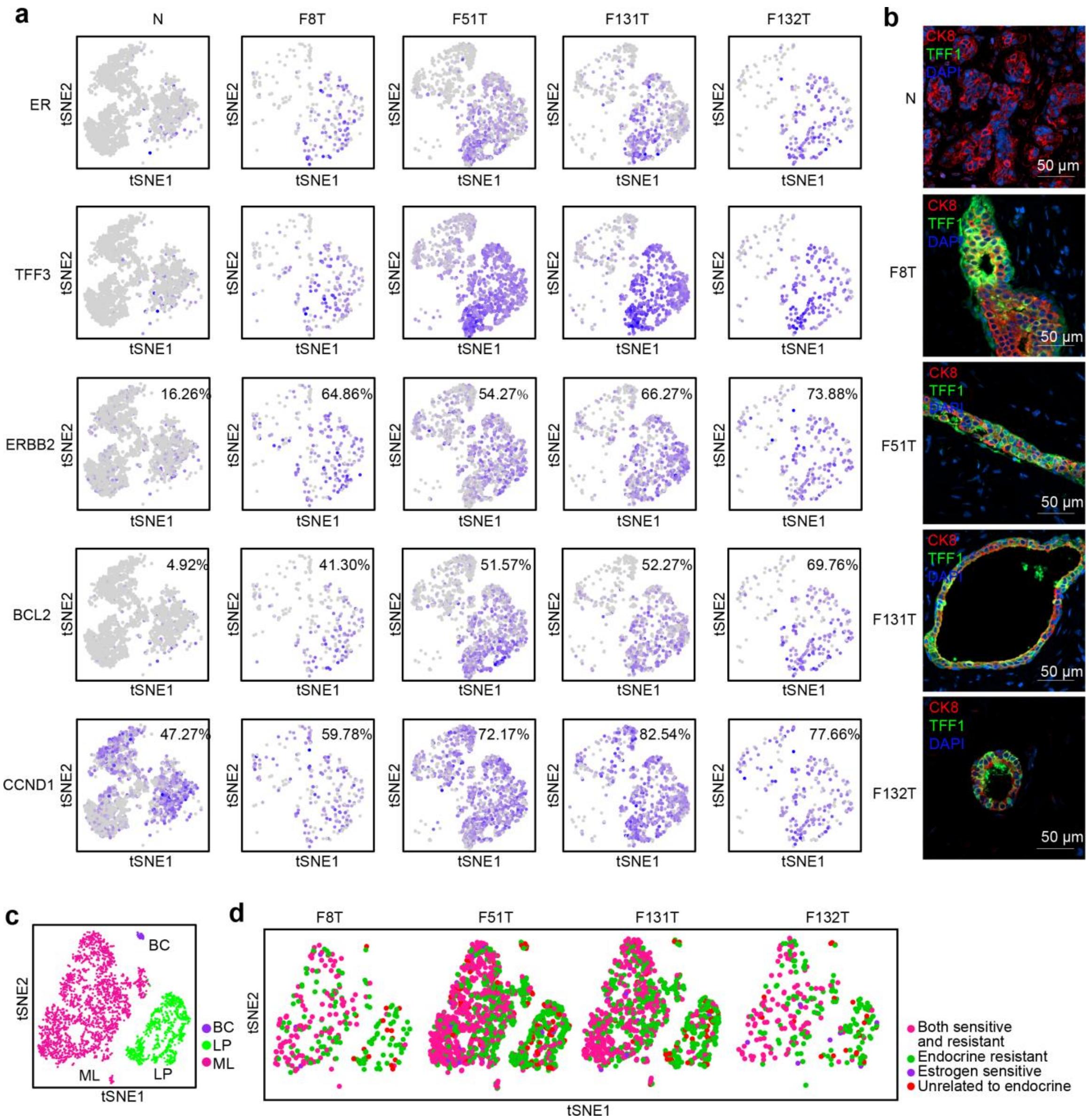

Supplementary Figure 10. Coexistence of hormone-sensitive and hormone-resistant fibroadenoma epithelial cells

a) Feature plot showing hormone-sensitive and hormone resistance gene expression in normal tissue and each fibroadenoma patient (3,173 from the normal; 276 from F8T, 1,592 from F51T, 1,014 from F131T, and 291 from F132T).

b) Representative immunofluorescence staining of TFF1 in each fibroadenoma and normal tissue (N). Scale bar: 50 µm. Experiment was performed with three independent replicates.

c, d) t-SNE clustering of 3,173 single-cell transcriptomes (276 from F8T, 1,592 from F51T, 1,014 from F131T, and 291 from F132T) colored by significant cell type clusters and split by patient.

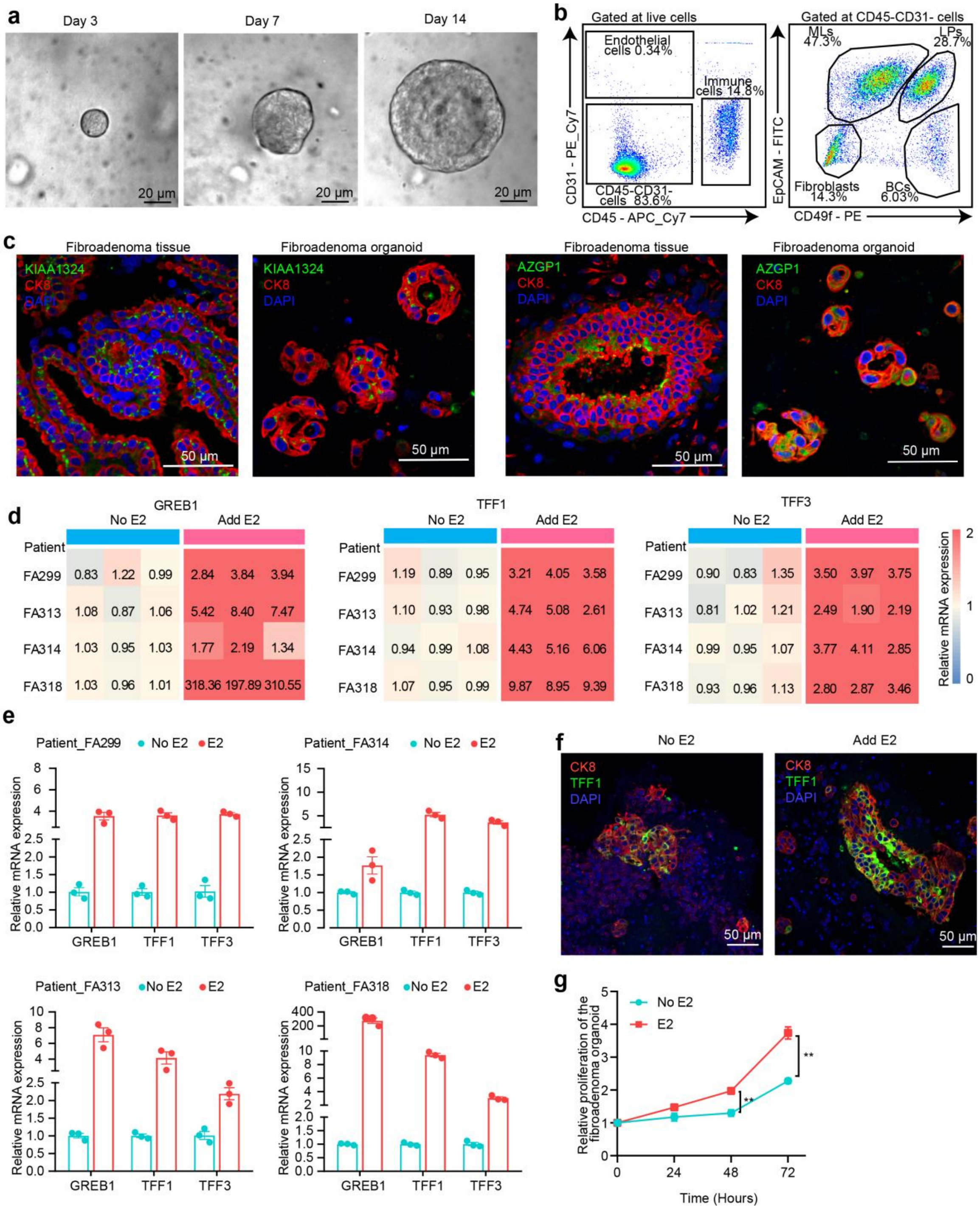

Patient	GREB1 No E2			GREB1 Add E2			TFF1 No E2			TFF1 Add E2			TFF3 No E2			TFF3 Add E2		
FA299	0.83	1.22	0.99	2.84	3.84	3.94	1.19	0.89	0.95	3.21	4.05	3.58	0.90	0.83	1.35	3.50	3.97	3.75
FA313	1.08	0.87	1.06	5.42	8.40	7.47	1.10	0.93	0.98	4.74	5.08	2.61	0.81	1.02	1.21	2.49	1.90	2.19
FA314	1.03	0.95	1.03	1.77	2.19	1.34	0.94	0.99	1.08	4.43	5.16	6.06	0.99	0.95	1.07	3.77	4.11	2.85
FA318	1.03	0.96	1.01	318.36	197.89	310.55	1.07	0.95	0.99	9.87	8.95	9.39	0.93	0.96	1.13	2.80	2.87	3.46

Supplementary Figure 11. Fibroadenoma organoids retain the structure and function of the human parental tissue

a) Bright-field images showing the generation of human breast fibroadenoma organoids. Scale bar: 20 μm. Experiment was performed with three independent replicates.

b) FACS analysis of cell components in fibroadenoma organoids, including fibroblasts (CD45-CD31- CD49f-EpCAM-), endothelial cells (CD31+) and immune cells (CD45+), basal cells (CD49fhiEpCAM-) and mature luminal cells (CD49floEpCAM+).
c) Immunofluorescence staining of characteristic markers in fibroadenoma tissue and organoids. Scale bar: 50 μm. Experiment was performed with three independent replicates.
d-e) Heatmap (d) and bar plot (e) showing the relative mRNA expression of estrogen responsive genes (*GREB1*, *TFF1*, *TFF3*) in the organoids with and without 10 nM estradiol (E2). Each value represents one technical replicate. FA299, FA313, FA314, FA318 represents 4 different organoids lines. Data in (e) are expressed as the mean +/- s.e.m.
f) Immunofluorescence staining of TFF1 in organoids with and without 10 nM estradiol (E2) treatment. Scale bar: 50 μm. Experiment was performed with three independent replicates.
g) Relative proliferation of fibroadenoma organoids with and without 10 nM estradiol (E2) treatment. n = 3/group from three independent experiments. Data are shown as mean +/− s.e.m. **p=0.0027 (48 h), ****p<0.0001 (72 h), two-way ANOVA. Source data are provided as a Source Data file.

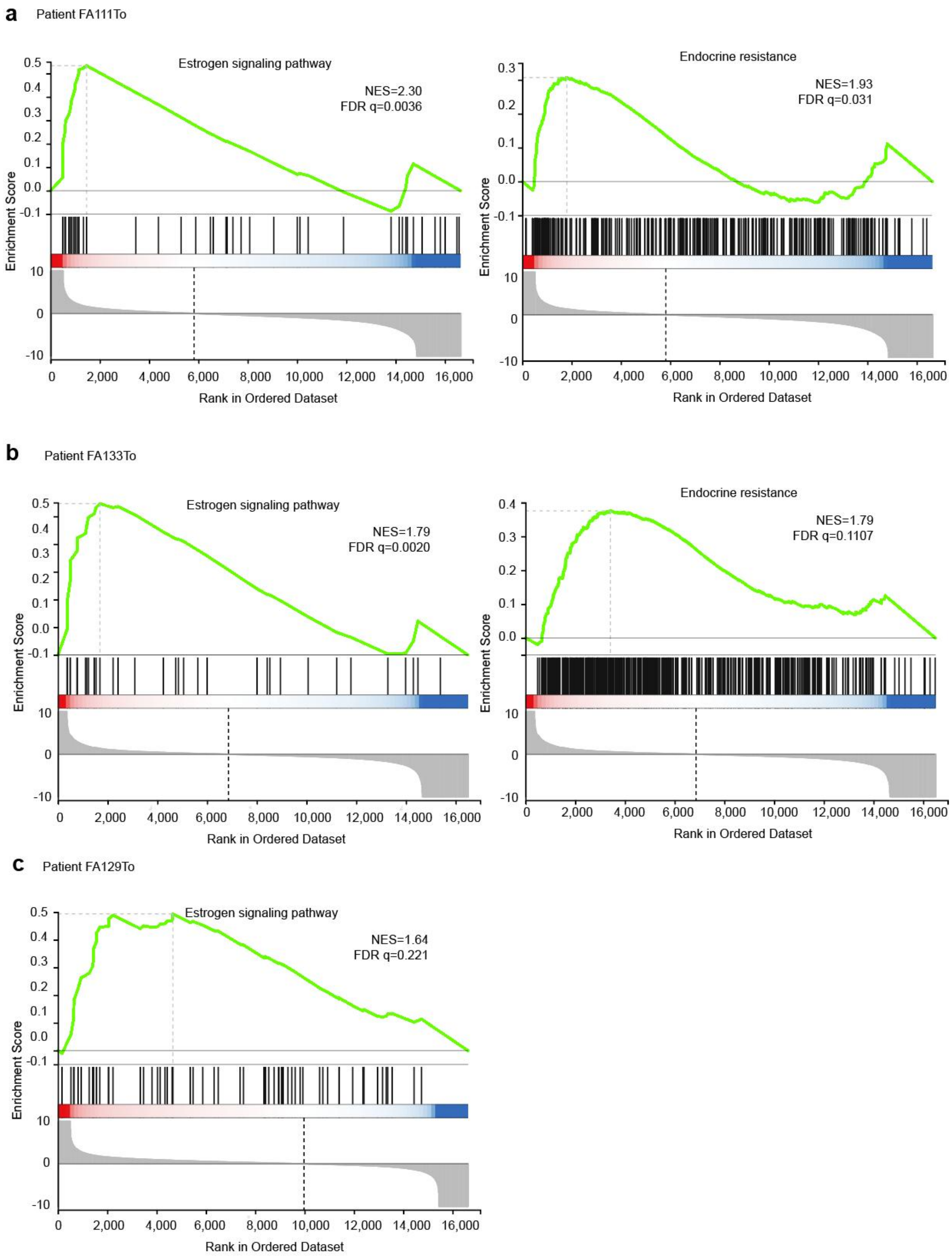

Supplementary Figure 12. ssGSEA of breast fibroadenoma organoids

a) ssGSEA of the estrogen signaling pathway and endocrine resistance in fibroadenoma versus normal organoids (F111To).

b) ssGSEA of the estrogen signaling pathway and endocrine resistance in fibroadenoma versus normal organoids (F133To).
c) ssGSEA of the estrogen signaling pathway in fibroadenoma versus normal organoids (F129To).

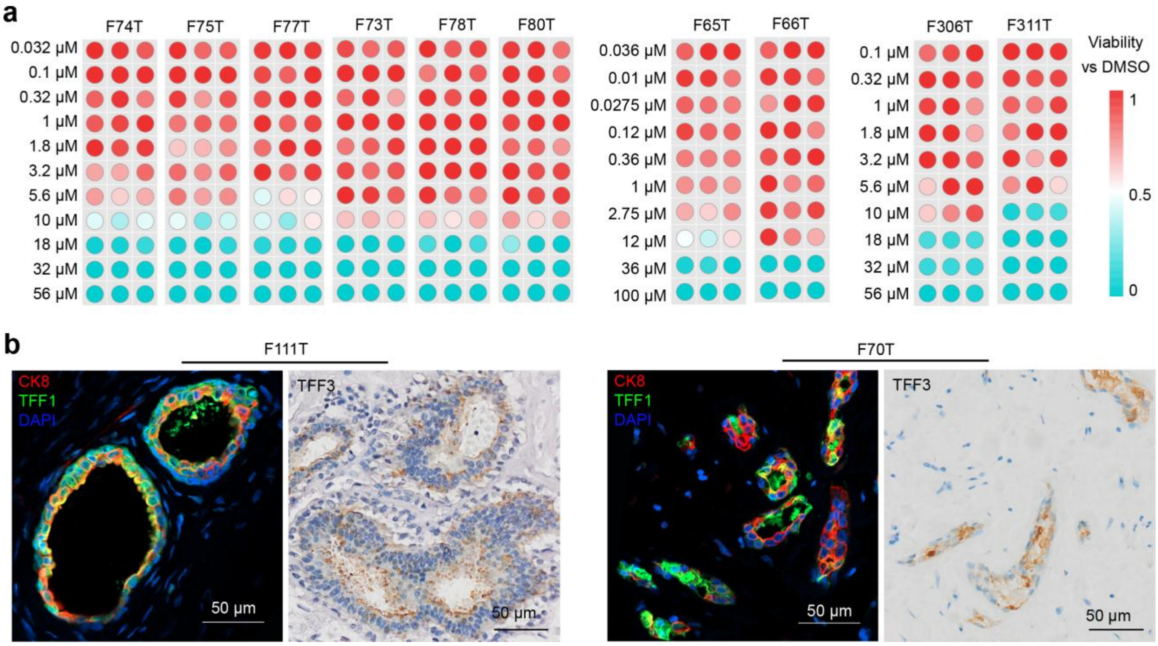

Supplementary Figure 13. Drug sensitivity of tamoxifen in breast fibroadenoma organoids

a) Heatmap of the drug sensitivity of tamoxifen in different fibroadenoma organoid lines.

b) Immunofluorescence staining of TFF1 and immunohistochemical staining for TFF3 in fibroadenoma tissues (F70T, F111T). Scale bar: 50 μm. Experiment was performed with three independent replicates.

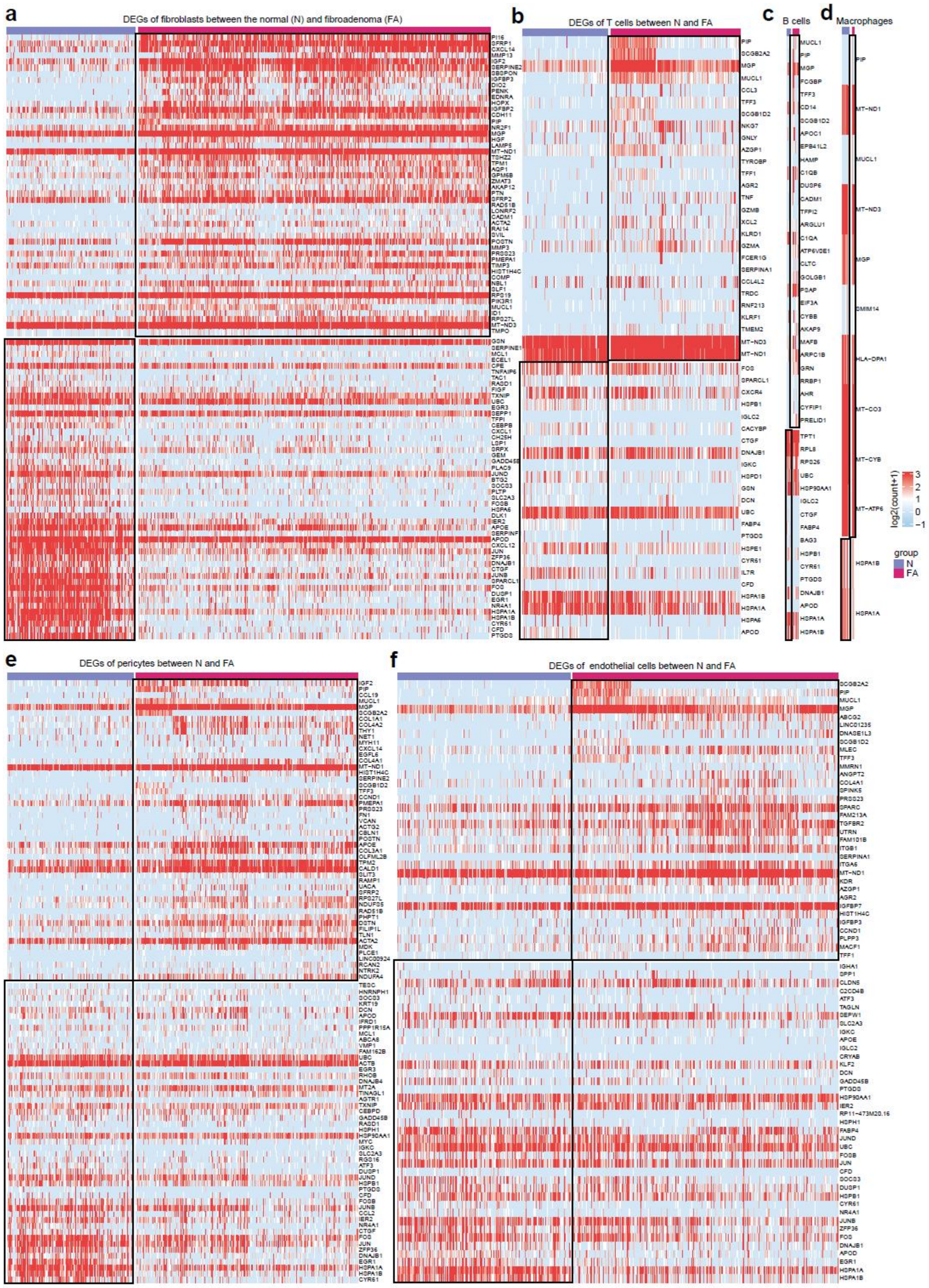

Supplementary Figure 14. DEGs of stromal cells, including fibroblasts, T cells, B cells, macrophages, pericytes, and endothelial cells, between fibroadenomas and normal tissues

a) Heatmap showing the distribution of upregulated and downregulated top DEGs identified in fibroblasts between fibroadenomas and normal tissues.

b) Heatmap showing the distribution of upregulated and downregulated top DEGs identified in T cells between fibroadenomas and normal tissues.

c) Heatmap showing the distribution of upregulated and downregulated top DEGs identified in B cells between fibroadenomas and normal tissues.

d) Heatmap showing the distribution of upregulated and downregulated top DEGs identified in macrophages between fibroadenomas and normal tissues.

e) Heatmap showing the distribution of upregulated and downregulated top DEGs identified in pericytes between fibroadenomas and normal tissues.

f) Heatmap showing the distribution of upregulated and downregulated top DEGs identified in endothelial cells between fibroadenomas and normal tissues.

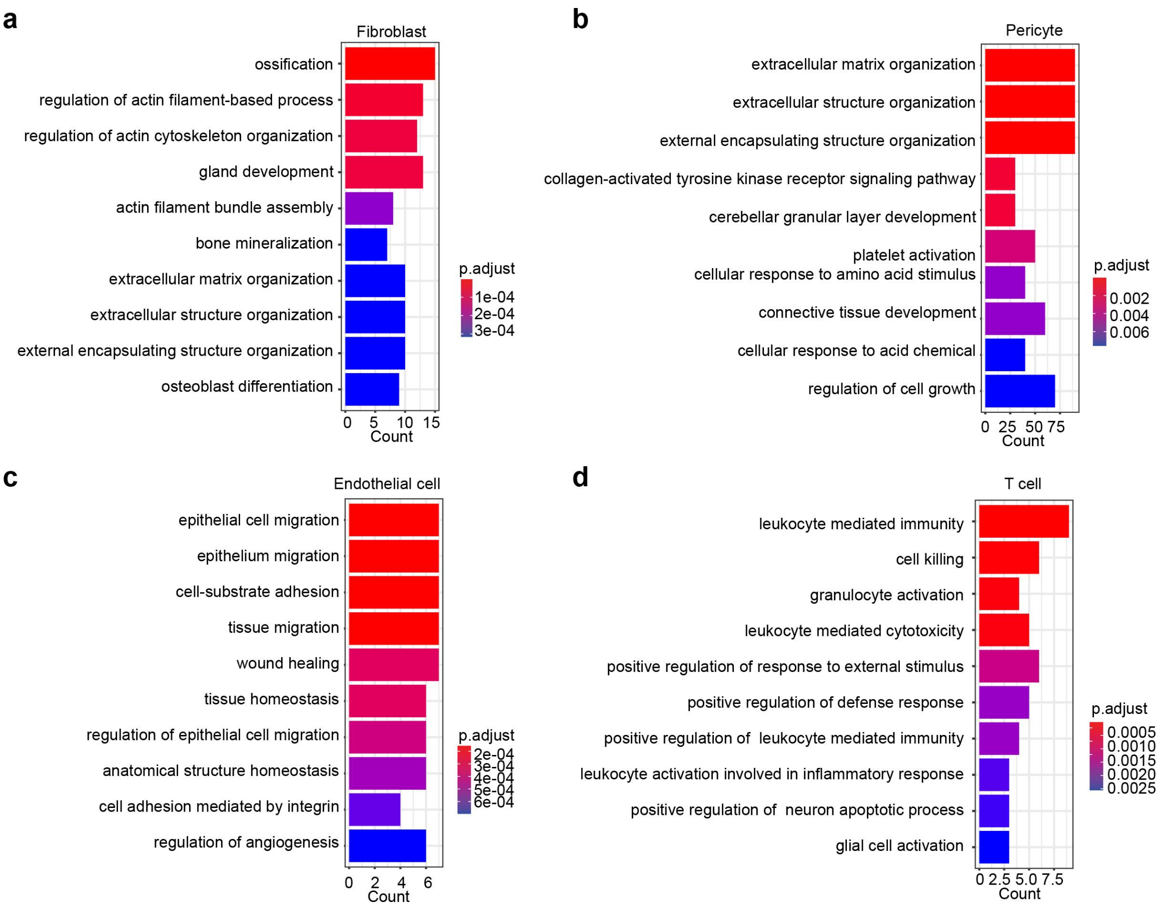

Supplementary Figure 15. GO enrichment analysis of DEGs in stromal cells between fibroadenomas and normal tissues

a) Representative GO terms of upregulated DEGs in fibroblasts between fibroadenomas and normal tissues. One-sided, hypergeometric test and adjusted by Benjamini-Hochberg correction.

b) Representative GO terms of upregulated DEGs in pericytes between fibroadenomas and normal tissues. One-sided, hypergeometric test and adjusted by Benjamini-Hochberg correction.

c) Representative GO terms of upregulated DEGs in endothelial cells between fibroadenomas and normal tissues. One-sided, hypergeometric test and adjusted by Benjamini-Hochberg correction.

d) Representative GO terms of upregulated DEGs in T cells between fibroadenomas and normal tissues. One-sided, hypergeometric test and adjusted by Benjamini-Hochberg correction.

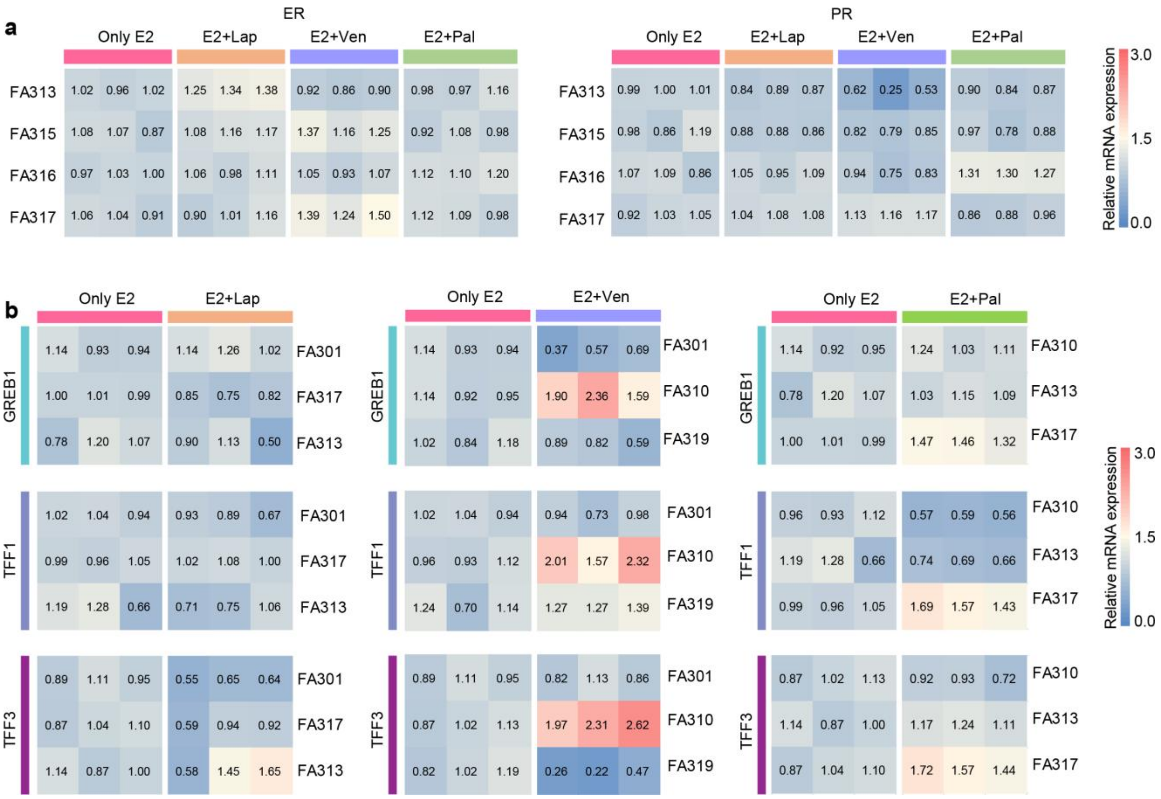

Supplementary Figure 16. The effect of resistance gene inhibitors on the expression of hormone receptor and estrogen target genes in fibroadenoma.

a) Heatmap showing the relative mRNA expression of hormone receptors (*ER*, *PR*) in organoids with and without treatment with 10 nM CDK4/6 inhibitor palbociclib (Pal), 5 μM BCL2 inhibitor venetoclax (Ven) and 10 nM ERBB2 inhibitor lapatinib (Lap). Each value represents one technical replicate. FA313, FA315, FA316, FA317 represents different organoids lines.

b) Heatmap showing the relative mRNA expression of estrogen responsive genes (*GREB1*, *TFF1*, *TFF3*) in the organoids with and without treatment with CDK4/6 inhibitor palbociclib (Pal) 10 nM, BCL2 inhibitor venetoclax (Ven) 5 μM and the ERBB2 inhibitor lapatinib (Lap) 10 nM. Each value represents one technical replicate. FA301, FA310, FA313, FA316, FA317 and FA319 represents different organoids lines. Source data are provided as a Source Data file.

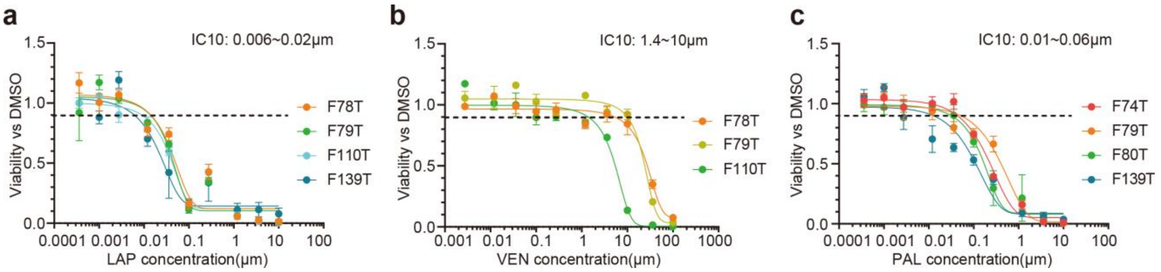

Supplementary Figure 17. Drug sensitivity of CDK4/6 inhibitor palbociclib (Pal), BCL2 inhibitor venetoclax (Ven) and ERBB2 inhibitor lapatinib (Lap) on human fibroadenoma organoids.

a-c) Drug sensitivity of lapatinib (Lap) (a), venetoclax (Ven) (b) and palbociclib (Pal) (c) in different fibroadenoma organoid lines. Each dot indicates a value from one experiment and n = 3/group from three independent experiments. F78T, F79T, F80T, F110T and F139T represents different organoids lines. Data are shown as mean +/− s.e.m. Source data are provided as a Source Data file.

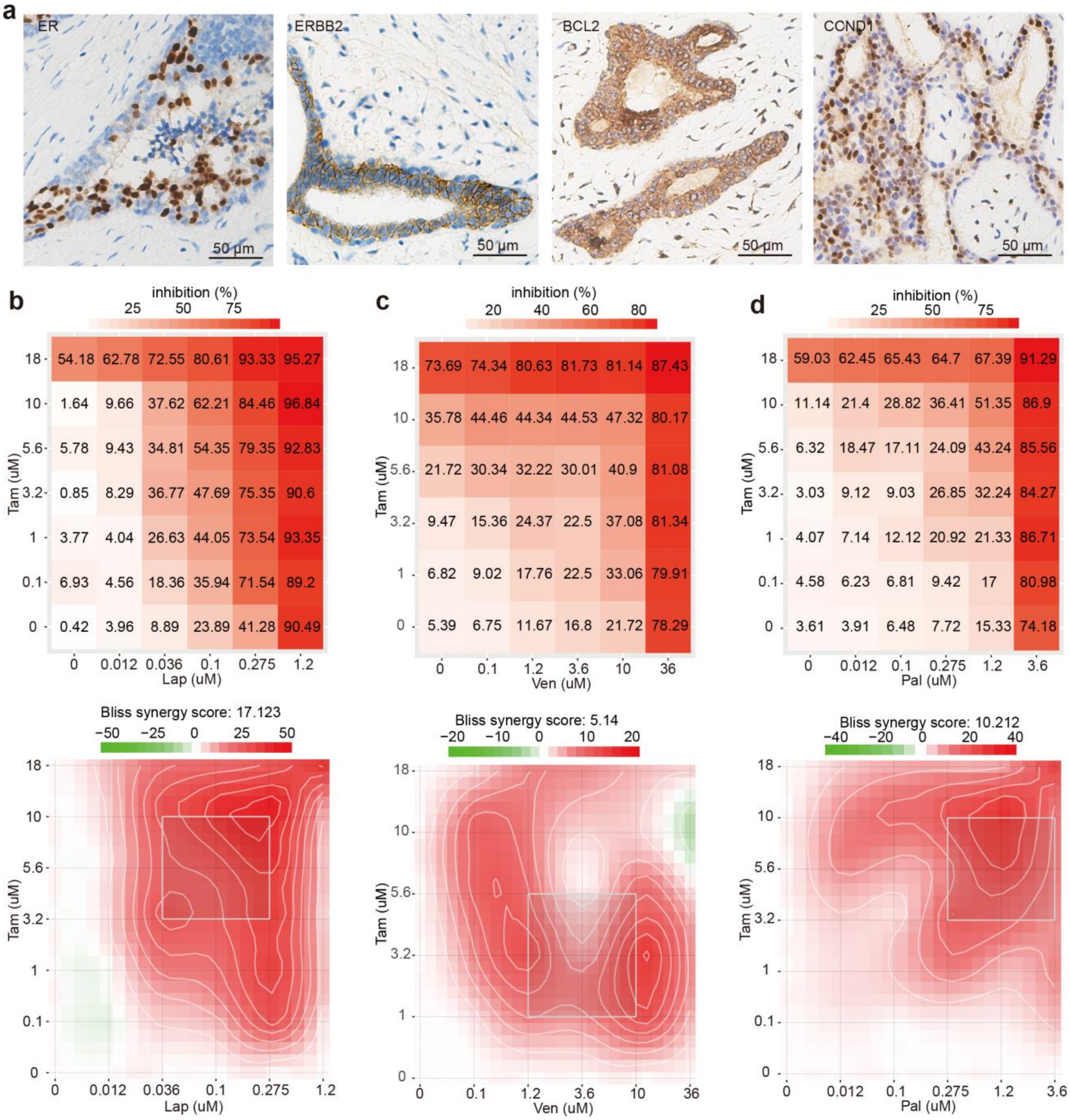

Supplementary Figure 18. Synergy analysis between tamoxifen and CDK4/6 inhibitor palbociclib (Pal), BCL2 inhibitor venetoclax (Ven) and ERBB2 inhibitor lapatinib (Lap) on human fibroadenoma organoids.

a) IHC of resistance pathway markers (ERBB2, BCL2, CCND1) on patient fibroadenoma tissues(F400). Scale bar: 50 μm. ERBB2: 2+(high expression); BCL2: H score=180(low expression); CCND1: H score=220(high expression). Experiment was performed with three independent replicates.

b-d) Dose-response matrices (inhibition) and synergy distributions of tamoxifen combined with resistant pathway inhibitors, including lapatinib (Lap) (b), venetoclax (Ven) (c) and palbociclib (Pal) (d). Source data are provided as a Source Data file.

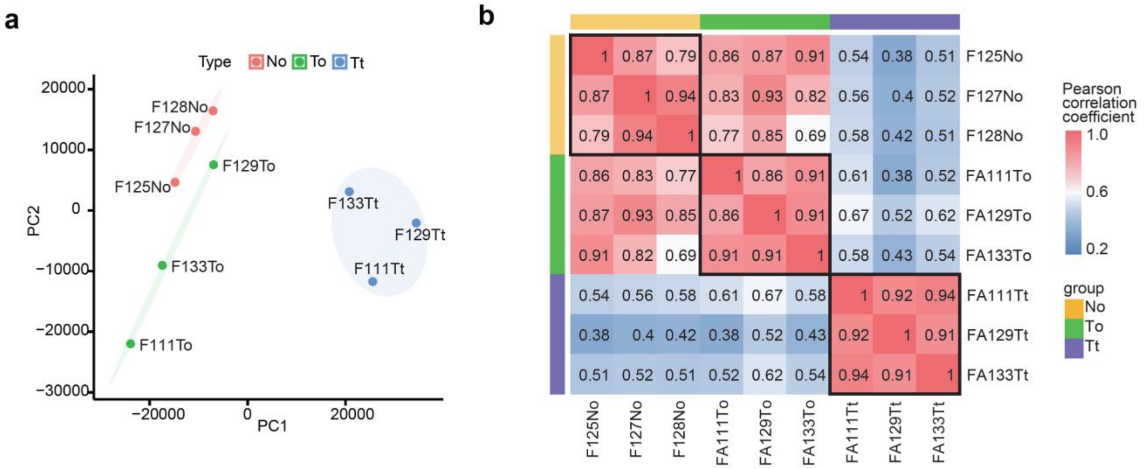

	F125No	F127No	F128No	FA111To	FA129To	FA133To	FA111Tt	FA129Tt	FA133Tt
F125No	1	0.87	0.79	0.86	0.87	0.91	0.54	0.38	0.51
F127No	0.87	1	0.94	0.83	0.93	0.82	0.56	0.4	0.52
F128No	0.79	0.94	1	0.77	0.85	0.69	0.58	0.42	0.51
FA111To	0.86	0.83	0.77	1	0.86	0.91	0.61	0.38	0.52
FA129To	0.87	0.93	0.85	0.86	1	0.91	0.67	0.52	0.62
FA133To	0.91	0.82	0.69	0.91	0.91	1	0.58	0.43	0.54
FA111Tt	0.54	0.56	0.58	0.61	0.67	0.58	1	0.92	0.94
FA129Tt	0.38	0.4	0.42	0.38	0.52	0.43	0.92	1	0.91
FA133Tt	0.51	0.52	0.51	0.52	0.62	0.54	0.94	0.91	1

Supplementary Figure 19. Transcriptome correlations of organoids compared to human fibroadenoma tissues.

a) PCA analysis (principal component analysis) of gene expression across the entire transcriptome for the the normal epithelium (No), the FA organoid (To) and the FA tissue (Tt) samples.

b) Quantitative transcriptome correlations are shown using a heatmap of Pearson correlations for gene-level log10-transformed RNA-Seq.
