# Supplementary material for: Single cell profiling of female breast fibroadenoma reveals distinct epithelial cell compositions and therapeutic targets
Source: Nat Commun. 2023 Jun 16;14:3469. doi: 10.1038/s41467-023-39059-3 (PMC10275980; doi:10.1038/s41467-023-39059-3)
Supplement: Supplementary file 2 — Reporting Summary [file 41467_2023_39059_MOESM2_ESM.pdf]

nature portfolio

Corresponding author(s): Jian Huang

Last updated by author(s): May 3, 2023

Reporting Summary

Nature Portfolio wishes to improve the reproducibility of the work that we publish. This form provides structure for consistency and transparency in reporting. For further information on Nature Portfolio policies, see our Editorial Policies and the Editorial Policy Checklist.

Statistics

For all statistical analyses, confirm that the following items are present in the figure legend, table legend, main text, or Methods section.

n/a	Confirmed	
☐	☒	The exact sample size (*n*) for each experimental group/condition, given as a discrete number and unit of measurement
☐	☒	A statement on whether measurements were taken from distinct samples or whether the same sample was measured repeatedly
☐	☒	The statistical test(s) used AND whether they are one- or two-sided<br>*Only common tests should be described solely by name; describe more complex techniques in the Methods section.*
☒	☐	A description of all covariates tested
☐	☒	A description of any assumptions or corrections, such as tests of normality and adjustment for multiple comparisons
☐	☒	A full description of the statistical parameters including central tendency (e.g. means) or other basic estimates (e.g. regression coefficient) AND variation (e.g. standard deviation) or associated estimates of uncertainty (e.g. confidence intervals)
☐	☒	For null hypothesis testing, the test statistic (e.g. *F*, *t*, *r*) with confidence intervals, effect sizes, degrees of freedom and *P* value noted<br>*Give P values as exact values whenever suitable.*
☒	☐	For Bayesian analysis, information on the choice of priors and Markov chain Monte Carlo settings
☐	☒	For hierarchical and complex designs, identification of the appropriate level for tests and full reporting of outcomes
☒	☐	Estimates of effect sizes (e.g. Cohen's *d*, Pearson's *r*), indicating how they were calculated

Our web collection on statistics for biologists contains articles on many of the points above.

Software and code

Policy information about availability of computer code

Data collection	scRNA-seq: 10X Chromium Single Cell Platform (10x Genomics Single Cell 3' v3 kit), Illumina NovaSeq 6000 (Illumina); Flow cytometry: FACSCanto II flow cytometer and FACSAria III cell sorter (BD Biosciences); Immunostaining acquisition: LSM800 (Carl Zeiss), VS200 (Olympus); Real-Time PCR: Applied Biosystem 7500 instrument (Applied Biosystems); Limited RNA-seq: MGISEQ-2000 platform (BGI); Whole-exome sequencing: DNBSEQ platform (BGI); Drug test: SpectraMax® iD3 multifunction reader (Molecular Devices).
Data analysis	Flow cytometry: FlowJo software (version 10.0); Immunostaining: imageJ2, ZEN 3.4 (blue edition) , Olympus Image Viewer 3; scRNA-seq data analysis: CellRanger (version 4.0.0, 10x Genomics) (https://github.com/10XGenomics/cellranger), Seurat R packages (V 3.2.0) (http://satijalab.org/seurat/); Limited RNA-seq: HISAT2 (v2.0.4) (http://daehwankimlab.github.io/hisat2/), Bowtie2 (v2.2.5) (https://bowtie-bio.sourceforge.net/bowtie2/index.shtml) and RSEM (v1.2.12) (http://deweylab.github.io/RSEM/); Whole-exome sequencing: BWA(v0.7.17) (https://bio-bwa.sourceforge.net/), GATK4 (v4.1.4.1) (https://gatk.broadinstitute.org/hc/en-us) and Snpeff(5.1)(http://pcingola.github.io/SnpEff/). Bliss independence model for synergy score: SynergyFinder web application (version 3.0).

For manuscripts utilizing custom algorithms or software that are central to the research but not yet described in published literature, software must be made available to editors and reviewers. We strongly encourage code deposition in a community repository (e.g. GitHub). See the Nature Portfolio guidelines for submitting code & software for further information.

Data

Policy information about availability of data

All manuscripts must include a data availability statement. This statement should provide the following information, where applicable:

- Accession codes, unique identifiers, or web links for publicly available datasets
- A description of any restrictions on data availability
- For clinical datasets or third party data, please ensure that the statement adheres to our policy

The raw single-cell RNA sequencing data, limited RNA sequencing data and whole exome sequencing data generated in this study have been deposited in the Genome Sequence Archive (GSA) (https://ngdc.cncb.ac.cn/gsa/) in the National Genomics Data Center, China National Center for Bioinformation/Beijing Institute of Genomics, Chinese Academy of Sciences, under accession number HRA002242 (https://ngdc.cncb.ac.cn/gsa-human/request/HRA002242). All data are available under restricted access, access can be obtained by completing the online data access request process after registration in the NGDC system as an approved user. The remaining data generated in this study are provided in the Supplementary Information/Source Data file. The single-cell datasets of normal human mammary epithelial cells used in this study are available in the NCBI database under accession code GSE113197 (https://www.ncbi.nlm.nih.gov/pmc/articles/PMC5966421/).

Field-specific reporting

Please select the one below that is the best fit for your research. If you are not sure, read the appropriate sections before making your selection.

- [x] Life sciences
- [ ] Behavioural & social sciences
- [ ] Ecological, evolutionary & environmental sciences

For a reference copy of the document with all sections, see nature.com/documents/nr-reporting-summary-flat.pdf

Life sciences study design

All studies must disclose on these points even when the disclosure is negative.

Sample size	In our study, a total of 135 patient samples were included. For scRNA-seq analysis, we included 4 patient samples and 2 normal samples from our data, as well as four single-cell datasets of normal human mammary epithelial cells downloaded from the NCBI database GSE113197 (https://www.ncbi.nlm.nih.gov/pmc/articles/PMC5966421/) due to the difficulty in obtaining enough paired normal tissues. And a total of 28,101 cells, with 17,776 from fibroadenoma (F8T: 5,297, F51T: 4,389, F131T: 3,980, F132T: 4,110) and 10,325 from normal tissue (F8N: 3,194, F51N: 7,131) was included to identify the total landscape, while a total of 6,346 epithelial cells, split evenly between fibroadenoma (F8T: 276, F51T: 1,592, F131T: 1,014, F132T: 291) and normal cells (F8N: 138, F51N: 173, Ind4: 485, Ind5: 830, Ind6: 710, Ind7: 837) was included to evaluate the compositions of epithelium. FACS was performed on 59 fibroadenoma samples and 21 normal samples. For immuno-staining, sections from 60 patient were included in the study. In vitro co-culture experiment was performed on 3 patients. To compare the relapse and unrelapse, 24 patients (relapse: 15; unrelapse: 9) were included. To compare the fibroadenoma component and the cancer component, 8 patients who underwent carcinoma pathologically proven to be derived from fibroadenoma were included. Sample size both for in vitro and in vivo was chosen taking in consideration the means of the target values between the experimental group and the control group, the standard error and the statistical analysis used. The sample size was able to ensure that our results were reliable.
Data exclusions	For single-cell RNA-sequencing data, we discard low-quality genes and cells according to the number of expressed genes, UMIs and mitochondrial reads ratio. For limited RNA sequencing data, we excluded base according to low-quality base ratio and unknown base ('N' base) ratio. For Whole-exome sequencing data, we removed reads of low quality, joint contamination or high proportion of N-bases. The details could be checked in Methods.
Replication	All replications were successful, the details could be checked in figure legends.
Randomization	Single cell RNA sequencing of epithelial cells were sampled randomly. The patients with fibroadenoma were recruited randomly except for the relapsed and unrelapsed, cancer derived from fibroadenoma as well as fibroadenoma which was clinically resistant. For the comparation of the relapse and unrelapse, we selected 15 patients with FAs who experienced relapse in the same breast location within 3 years after surgery and 9 patients without relapse by retrospectively collecting clinical data. For the comparation of the fibroadenoma component and the cancer component, we selected 8 patients who underwent carcinoma pathologically proven to be derived from fibroadenoma.
Blinding	Blinding was only performed in IHC score calculation of CCND1 as well as BCL2 and grade estimation of ERBB2 in fig3d, e and table 1. The investigators were blinded to group allocation during data collection and/or analysis. Blinding was not applicable in other experiment since it is exploratory in character and have no elements that might be influenced by bias from the subject or observer.

Reporting for specific materials, systems and methods

We require information from authors about some types of materials, experimental systems and methods used in many studies. Here, indicate whether each material, system or method listed is relevant to your study. If you are not sure if a list item applies to your research, read the appropriate section before selecting a response.

Materials & experimental systems

n/a	Involved in the study
[ ]	[x] Antibodies
[x]	[ ] Eukaryotic cell lines
[x]	[ ] Palaeontology and archaeology
[x]	[ ] Animals and other organisms
[ ]	[x] Human research participants
[x]	[ ] Clinical data
[x]	[ ] Dual use research of concern

Methods

n/a	Involved in the study
[x]	[ ] ChIP-seq
[ ]	[x] Flow cytometry
[x]	[ ] MRI-based neuroimaging

Antibodies

Antibodies used

Antibodies used for FACS:
CD45 (Biolegend, Clone:HI30, Cat.# 304014, [1:100]);
CD31 (Biolegend, Clone: WM59, Cat.# 303118, [1:100]);
EpCAM(Biolegend, Clone: 9C4, Cat.# 324204, [1:100]);
CD49f (Biolegend, Clone: GoH3,Cat.# 313612, [1:100]).

Antibodies used for immunostaining:
ER (Leica, clone: 6F11, Cat.# PA0151, [1:600]);
PR(Leica, clone: 16, Cat.# PA0312, [1:600]);
ERBB2 (Roche, clone: 4B5, Cat.# 05999570001, [1:1]);
BCL2(ZSGB-BIO, clone: EP36,Cat.#ZA-0536, [1:400]);
CCND1(ZSGB-BIO, clone: SA38-08, Cat.# ZA-0101, [1:800]);
TFF3(Abcam, clone: EPR3974, Cat.# ab108599, [1:500]);
CK8 (Hangzhou HuaAn Biotechnolofy, colon: A1-B11, Cat.# M1603-2, [1:100]);
CK14 (Abcam, colon: EP1612Y, Cat.# ab51054, [1:100]);
PRLR (Abcam, clone: EPR7184(2), Cat.# ab170935, [1:100];
TFF1 (Abcam, Clone: EPR3972, Cat.# ab92377, [1:100]);
KIAA1324 (Novus, Cat.# NBP2-57699, [1:500]).

Secondary antibodies:
Goat pABs to Rb IgG (Alexa Fluor® 488) (Abcam, Cat.# ab150081, [1:200]);
Goat pABs to Ms IgG (Alexa Fluor® 555) (Abcam, Cat.# ab150118, [1:200]);
horseradish peroxidase conjugated anti-mouse&rabbit (absin, Cat.# 996, [1:200]).

Validation

All antibodies used for FACS (CD45-APC_Cy7, CD31-PE_Cy7, EpCAM-FITC and CD49f-PE) were validated by the manufacturer and widely cited in the literature.

ER staining was nuclear as expected.
PR staining was nuclear as expected.
The TFF3 antibody was validated via knockdown by the manufacturer and 18 publications using this antibody.
The CK14 antibody was validated via knockdown by the manufacturer and 16 publications using this antibody.
Antibody staining of PRLR was stained as expected. Used in 6 citations.
Antibody staining of TFF1 was stained as expected. Used in 18 citations.
Antibody staining of CK8 was stained as expected. Used in 2 citations.
KIAA1324 staining was cytoplasmic as expected.
ERBB2 staining was cytoplasmic as expected.
BCL2 staining was cytoplasmic as expected.
CCND1 staining was cytoplasmic as expected.

Human research participants

Policy information about studies involving human research participants

Population characteristics

The patients included in this study, with the exception of cancer derived from fibroadenoma (34-62y), were premenopausal women aged 19 to 49 years. Clinical information is summarized in Table S1.

Recruitment

Human tissues were collected from the Second Affiliated Hospital of Zhejiang University, School of Medicine.

Ethics oversight

The collection of removed tissues were approved by the ethics review committee of the Second Affiliated Hospital of Zhejiang University School of Medicine (I2021001303).

Note that full information on the approval of the study protocol must also be provided in the manuscript.

Flow Cytometry

Plots

Confirm that:

- [x] The axis labels state the marker and fluorochrome used (e.g. CD4-FITC).
- [x] The axis scales are clearly visible. Include numbers along axes only for bottom left plot of group (a 'group' is an analysis of identical markers).
- [x] All plots are contour plots with outliers or pseudocolor plots.
- [x] A numerical value for number of cells or percentage (with statistics) is provided.

Methodology

Sample preparation	Tissues were cut thoroughly by scissors and plated in advanced DMEM/F12 (Sigma Life Science, 12634010) medium containing 1 mg/ml collagenase (Sigma Aldrich, Cat.# C9407) in gentleMACS c-tubes (Miltenyi Biotec, 130–096-334). Samples were dissociated completely by a gentleMACS octo Dissociator with Heaters (Miltenyi Biotec, Germany) for 1 h. The digested tissue suspension was strained over a 100 µm filter and pelleted at 400 g for 10 min. Then, erythrocytes were lysed in 2 mL red blood cell lysis buffer (BOSTER, AR1118) for 5 min at room temperature.
Instrument	All FACS analysis were performed on a FACSCanto II flow cytometer, and all sorting were performed on a FACSAria III cell sorter.
Software	FlowJo software (V10.0)
Cell population abundance	The abundance of the relevant cell subset sorted by FACS reached >99% detected on a FACSCanto II flow cytometer.
Gating strategy	The gating strategy used in figure 1g, figure 4f, Extended Data Figure 11b and sample preparation of figure 4d, e has been done as it follows: SSC-A/FSC-A, FSC-H/FSC-A, SSC-H/SSC-A, FSC-A/Zombie Red (Live cell), CD31/CD45 (CD31-CD45- cell, CD31+ cell and CD45+ cell), EpCAM/CD49f(EpCAM-CD49f lo cell, EpCAM+CD49f lo cell, EpCAM+CD49f hi cell and EpCAM-CD49f hi cell). Further information are displayed in the Supplementary figure 5.

- [x] Tick this box to confirm that a figure exemplifying the gating strategy is provided in the Supplementary Information.
